# In the moonlight: non-catalytic functions of ubiquitin and ubiquitin-like proteases

**DOI:** 10.3389/fmolb.2024.1349509

**Published:** 2024-02-22

**Authors:** Marta Campos Alonso, Klaus-Peter Knobeloch

**Affiliations:** ^1^ Institute of Neuropathology, Faculty of Medicine, University of Freiburg, Freiburg, Germany; ^2^ CIBSS—Centre for Integrative Biological Signalling Studies, University of Freiburg, Freiburg, Germany

**Keywords:** deubiquitinase, ubiquitin-like protease, non-catalytic function, non-hydrolytic function, non-enzymatic function, protein moonlighting, DUB, USP18

## Abstract

Proteases that cleave ubiquitin or ubiquitin-like proteins (UBLs) are critical players in maintaining the homeostasis of the organism. Concordantly, their dysregulation has been directly linked to various diseases, including cancer, neurodegeneration, developmental aberrations, cardiac disorders and inflammation. Given their potential as novel therapeutic targets, it is essential to fully understand their mechanisms of action. Traditionally, observed effects resulting from deficiencies in deubiquitinases (DUBs) and UBL proteases have often been attributed to the misregulation of substrate modification by ubiquitin or UBLs. Therefore, much research has focused on understanding the catalytic activities of these proteins. However, this view has overlooked the possibility that DUBs and UBL proteases might also have significant non-catalytic functions, which are more prevalent than previously believed and urgently require further investigation. Moreover, multiple examples have shown that either selective loss of only the protease activity or complete absence of these proteins can have different functional and physiological consequences. Furthermore, DUBs and UBL proteases have been shown to often contain domains or binding motifs that not only modulate their catalytic activity but can also mediate entirely different functions. This review aims to shed light on the non-catalytic, moonlighting functions of DUBs and UBL proteases, which extend beyond the hydrolysis of ubiquitin and UBL chains and are just beginning to emerge.

## 1 Introduction

Post-translational modifications (PTMs) significantly contribute to the increase in complexity from genome to proteome and regulate a myriad of cellular processes. One of the most important PTMs is ubiquitination, a process by which a 76 amino acid protein termed ubiquitin is covalently attached to thousands of substrates, impacting nearly all aspects of cellular functions.

Ubiquitin is encoded by four different genes (*UBA52*, *UBA80*, *UBB* and *UBC*) and is synthesized as an immature precursor protein whose C-terminal region undergoes processing to expose the characteristic diglycine motif of the mature form (Grou et al., 2015; Martín-Villanueva et al., 2021). Prior to its conjugation, ubiquitin is first activated at its C-terminal carboxyl group by an E1 enzyme in an ATP-dependent manner. Then, the ubiquitin molecule is transferred to an E2 enzyme, which cooperates with an E3 ligase to facilitate its attachment to the target substrate. Lastly and according to the classical ubiquitination process, the C-terminal carboxyl group of ubiquitin forms an amide bond with the ε-amino group of a lysine residue or the N-terminal methionine in the target protein (Damgaard, 2021). Although monoubiquitinations are frequent and play different roles within the cell, ubiquitin itself possesses seven lysines along with the amine group of its initial methionine residue that can themselves be subject to ubiquitination, generating chains of diverse size and linkages. This results in complex ubiquitination chains, commonly referred to as the “ubiquitin code” (Komander and Rape, 2012). Strikingly, different polyubiquitin linkages exhibit unique structures and thus, can exert various cellular functions by determining the fate and binding properties of modified substrates in different ways (Akutsu et al., 2016).

Notwithstanding, recent discoveries have further extended this classical ubiquitination paradigm by identifying novel forms of ubiquitination occurring on cysteine, serine and threonine substrate residues involving ester linkages. Moreover, while it was initially believed that only proteins could be ubiquitinated, data gathered over the last years clearly indicate that ubiquitination can also target lipids, sugars and nucleotides (reviewed in Dikic and Schulman, 2022; Kelsall, 2022; Sakamaki and Mizushima, 2023), increasing the complexity and the scope of this modification process.

Since the discovery of ubiquitin in 1975, diverse proteins with the same three-dimensional core structure, the β-grasp fold, have been uncovered and grouped under the designation of ubiquitin-like proteins (UBLs). Among the most studied are neural precursor cell expressed developmentally downregulated 8 (NEDD8), interferon-stimulated gene 15 (ISG15), human leukocyte antigen (HLA)-F adjacent transcript 10 (FAT10), small-ubiquitin-like modifier (SUMO), autophagy related modifiers ATG8 and ATG12, ubiquitin-fold modifier protein 1 (UFM1), ubiquitin-related modifier 1 (URM1) and monoclonal nonspecific suppressor factor β (MNSFβ, also known as FUBI) (Van Der Veen and Ploegh, 2012). Despite sharing structural similarities with ubiquitin, these proteins possess entirely distinct and unique functions, locations and regulatory mechanisms (Kerscher et al., 2006; Hochstrasser, 2009; Van Der Veen and Ploegh, 2012). Similar to ubiquitin, the majority of UBLs feature a C-terminal diglycine motif for isopeptide linkage to their target proteins through an enzymatic process that closely resembles ubiquitination. Notwithstanding, ATG8, ATG12 and UFM1 only have a single C-terminal glycine (Aichem and Groettrup, 2020). In addition, URM1 has been shown to form covalent bonds with various target proteins through a process that does not rely on E2/E3 enzymes (Ravichandran et al., 2022). Furthermore, numerous UBLs such as SUMO, ISG15, FAT10 and ATG12 have been demonstrated to perform indispensable functions by engaging with substrates in a non-covalent manner (Perng and Lenschow, 2018; Pang et al., 2019; Aichem and Groettrup, 2020; González-Prieto et al., 2021). Moreover, in contrast to ubiquitin, free ISG15 (Swaim et al., 2017) and fubiylated substrates (O’Dea et al., 2023) can be secreted into the extracellular space to perform additional functions. Nonetheless, despite their importance, most UBLs have been significantly less studied than ubiquitin and numerous aspects, including mechanisms of action and regulation, target recognition and cellular functions await to be explored.

Except for FAT10 (Aichem and Groettrup, 2020), for which no protease has been identified so far, the covalent modifications performed by ubiquitin and UBLs can be reversed by a group of enzymes commonly referred to as deubiquitinases (DUBs) and UBL proteases, respectively. Notably, beyond reversing substrate modifications, these proteins often process the C-terminal region of immature precursors of ubiquitin and UBLs (Ronau et al., 2016), which is a prerequisite for linkage.

In general, DUBs and UBL proteases can be classified in two different ways: based on the enzymatic process employed to cleave peptide bonds or according to the structure of their catalytic domain. In accordance with the first classification, these enzymes can be grouped into thiol proteases or metalloproteases, depending on whether they use cysteine or a metal element to perform their catalytic activity. Furthermore, these proteases can also be categorized into families based on the configuration of their catalytic region (Ronau et al., 2016). Accordingly, the 100 DUBs identified in humans are generally grouped into seven different families: ubiquitin carboxyl-terminal hydrolases (UCHs), ubiquitin-specific proteases (USPs), ovarian tumour domain proteins (OTUs), Machado-Joseph domain-containing proteases (MJDs), Jab1/Mov34/Mpr1Pad1 N-terminal domain proteases (JAMMs), motif interacting with Ub-containing novel DUB family (MINDYs) and zinc finger with UFM1-specific peptidase domain protein (ZUSFP) (Ronau et al., 2016; Clague et al., 2019). Recently, a new family of DUBs has been discovered, the Viral Tegument-like Deubiquitinases (VTD). However, to date, no members of this family have been identified in humans (Erven et al., 2022).

Due to the prevalence of a papain-like structure among the majority of DUBs families, where the catalytic cysteine residue is positioned at the N-terminal region of the core alpha helix (Ozhelvaci and Steczkiewicz, 2023), they are considered homologous and consequently are grouped together in the so called CA clan according to MEROPS database (Rawlings et al., 2018). Despite their mechanistic resemblances, UBL proteases usually possess a different structure characterized by a beta barrel subdomain containing the active site histidine and aspartate followed by a second subdomain consisting of a helical bundle that accommodates the catalytic cysteine (Rawlings and Barrett, 2013). Therefore, UBL proteases belong to separate families within the so called CE protease clan (Pruneda et al., 2016; Hermanns et al., 2018). For example, SUMO proteases can be categorized into three distinct families: SUMO/sentrin-specific proteases (SENPs), desumoylating isopeptidases (DESIs) and ubiquitin-specific protease-like 1 (USPL1) (Nayak and Müller, 2014; Ronau et al., 2016; Morrell and Sadanandom, 2019). Although the classification into the seven families mentioned above is the most commonly used, it has to be pointed out that names of DUBs and UBL proteases might be misleading in several cases. Examples include: (1) USP18, which despite its name (*Ubiquitin-specific protease 18*) does not recognize nor cleave ubiquitin but specifically deconjugates solely ISG15; (2) USP2, USP5, USP14 and USP21, which not exclusively cleave ubiquitinated substrates but also ISGylated proteins; (3) USP16 and USP36, which display cross-reactivity between ubiquitin, FUBI and ISG15 (Zhao et al., 2023) and (4) SENP8, which despite its name does not cleave SUMO at all but exhibits specificity for NEDD8 instead (Kunz et al., 2018).

Not only endogenous DUBs and UBL proteases can counteract ubiquitin/UBL modifications but also invading pathogens such as bacteria and viruses, which have developed numerous survival strategies to counteract the immune response of the organism by manipulating or undermining the host ubiquitin and ubiquitin-like modification systems. In particular, these pathogens have evolved an impressive arsenal of ligases and proteases. While most of pathogen derived ubiquitin/UBL modifying enzymes mimic the structures and functional motifs of their eukaryotic counterparts, some have developed entirely new and innovative mechanisms to hijack the host system (Isaacson and Ploegh, 2009; Randow and Lehner, 2009; Zhou and Zhu, 2015; Pruneda et al., 2016; Bailey-Elkin et al., 2017; Roberts et al., 2023). As an example, *Legionella pneumophila* has not only developed conventional DUBs and UBL proteases but also harbours enzymes capable of catalysing entirely new forms of ubiquitination such as phosphoribosylated-ubiquitination and transglutaminase-mediated ubiquitination. Furthermore, this pathogen has also evolved proteases that can reverse these modifications (reviewed in Kitao et al., 2020). The emergence and adaptation of such enzymes, driven by evolutionary pressures, can directly impact our society, as demonstrated by the recent pandemic caused by the SARS-CoV-2 virus. Here, the proteome of this virus contains a UBL protease known as papain-like protease (PLpro), which evolved to predominantly cleave ISG15 modifications, thereby undermining the human host immune response (Klemm et al., 2020; Shin et al., 2020). It is crucial to note that PLpro also plays a vital role in the replication of the virus by processing polyproteins nsp1, nsp2 and nsp3. Additionally, this protease also possesses the ability, albeit with low activity, to eliminate K48 polyubiquitin chains from substrates (Klemm et al., 2020).

DUBs and UBL proteases have a significant impact not only on pathogenic infections but also in maintaining homeostasis in the organism. Both the overexpression and loss-of-function mutations of genes encoding these proteins have been associated with various diseases, including cancer, neurodegeneration, developmental disorders, cardiac conditions and inflammation (Basar et al., 2021; Hwang et al., 2022). These observations, coupled with the recognition that DUBs and UBL proteases, akin to other enzymes, fall within the category of proteins amenable to small molecule inhibition, have sparked significant interest in exploring these proteases as novel targets for drug development and therapeutic intervention.

Even in the absence of a detailed analysis, it is often presumed that phenotypes arising from either the lack or altered levels of DUBs and UBL proteases result from disrupted ubiquitin/UBL deconjugation activities. However, beyond the catalytic core, most of these proteases contain additional domains that may not only modulate catalytic activity or target recognition but could also connect DUBs and UBL proteases to entirely different pathways, regardless of their hydrolytic activity. While this phenomenon has already been demonstrated in other enzymes (Rauch et al., 2011; Janisiw et al., 2020), the potential of DUBs and UBL proteases to mediate functions entirely unrelated to their protease activity is just beginning to emerge. Understanding how the catalytic and non-catalytic functions of these proteins are interconnected with their regulation, molecular processes and physiological functions is not only crucial for identifying pathomechanisms but also essential for developing appropriate strategies for therapeutic intervention. To achieve this, it is crucial to conduct mutagenesis studies to pinpoint specific motifs and residues involved in the different functions of DUBs and UBL proteases, thereby helping to unravel the molecular mechanisms at play. It is worth mentioning that while the traditional approach to study the catalytic activity of DUBs involves mutating the catalytic cysteine to alanine to suppress the hydrolysis of isopeptide bonds, such mutations can sometimes unexpectedly sequester cellular ubiquitin, leading to dominant negative effects unrelated to DUB activity loss. Alternatively, replacing the active cysteine with arginine can effectively inactivate DUBs and reduce their affinity for ubiquitin (Morrow et al., 2018). Despite being technically and financially more demanding, producing knock-in mice with targeted mutations in key residues in the different domains of these proteases would be immensely beneficial for evaluating the physiological effects of their various functions.

In this review we want to give visibility to often unexpected, non-catalytic, so called “moonlighting” functions of DUBs and UBL proteases, which can at times be essential for proper functioning of the organism and highly relevant for physiological processes or pathological alterations. By this, we mean to highlight functions performed by these proteins that extend beyond the hydrolysis of ubiquitin and UBL immature precursors and chains. Despite the limited tools currently available to study these non-catalytic activities, a comprehensive analysis has the potential to ascribe novel functions to DUB and UBL proteases, provide fresh insights into disease mechanisms and advance therapeutic possibilities.

## 2 Catalytic activities of DUBs and UBLs proteases

As mentioned above, ubiquitin molecules themselves can undergo ubiquitination, resulting in the formation of polyubiquitinated substrates. The nature of these polymeric chains can be categorized as either homotypic or heterotypic, depending on whether the modified ubiquitin residue remains consistent or varies. Substrates modified with homotypic chains have been found to be involved in many but different cellular functions: innate immunity (K27, K33, K63 and M1), DNA damage response (K6 and K27), proteasomal degradation (K11 and K48), protein trafficking (K33 and K63), mitophagy (K6), Wnt/β-catening signalling (K29) and inflammatory nuclear factor-κB (NF-κB) signalling (M1) (Akutsu et al., 2016; Swatek and Komander, 2016; van Wijk et al., 2019; Tracz and Bialek, 2021). Heterotypic chains can be further classified as mixed or branched, depending on whether ubiquitin molecules are modified at a single residue or multiple ones. While homotypic chains have been studied extensively, structures and functions of heterotypic chains remain ill defined (reviewed in French et al., 2021; Kolla et al., 2022). Moreover, ubiquitin can form hybrid chains with other UBL molecules such as SUMO, NEDD8 or ISG15. However, the cellular functions of such polymers are largely elusive (Pérez Berrocal et al., 2020). The extensive variety of ubiquitin polymers not only significantly enhances the intricacy of the signals but also expands the range of biological information they can convey. To compound this complexity, numerous residues within ubiquitin molecules can be subject to phosphorylation and acetylation, adding further complexity to the system (Swatek and Komander, 2016).

This extensive array of chains can be recognized by different DUBs. The selectivity and specificity of DUBs are determined by the characteristics of the distinct binding sites that accommodate the side chains of the amino acid residues of ubiquitin and/or the substrate. These binding sites are arranged along the groove containing the active site of the protease, which is responsible for the hydrolysis of the scissile bond. They are numbered from the catalytic site as S1, S2 … Sn towards the N-terminal end of the substrate, and as S1’, S2’…Sn’ towards the C-terminal region (Schechter and Berger, 1967). Initially, these enzymes identify an ubiquitin moiety at their S1 site primarily through hydrophobic interactions with the isoleucine 44 patch of ubiquitin. This frequently leads to conformational changes in the protease to position the scissile bond within its active site for cleavage. Most DUBs additionally have an S1’ site in their catalytic domain or in an auxiliar domain to which the ubiquitin molecule following the scissile bond binds. These interactions can be decisive in establishing the linkage type that a given DUB can recognize. DUBs lacking this pocket may instead have a substrate-specific S1’ binding site. Besides, some DUBs are able to discriminate between different chain lengths and/or linkage type due to the presence of supplementary ubiquitin binding sites within their catalytic domain or in the so-called ubiquitin-binding domains (UBDs) (Flierman et al., 2016; Mevissen and Komander, 2017; Lange et al., 2022).

In contrast to the wealth of data on ubiquitin chain formation, our understanding on polymer formation by UBLs remains quite limited. Among the most studied is SUMO, whose three paralogs, namely, SUMO-1, SUMO-2 and SUMO-3, have been observed to undergo polymerization both *in vitro* and *in vivo* albeit in different ways (Matic et al., 2008). SUMO-2 and SUMO-3 are virtually identical and can form polymeric chains via the lysine residue at position 11 within their SUMOylation consensus site. In contrast, SUMO-1 has an inverted SUMO motif that does not contain any lysine and, consequently, although it can SUMOylate substrates, is unable to form chains with other SUMO molecules. Nonetheless, there is evidence suggesting that SUMO-1 can act as a “stopper”, terminating the polymeric chains formed by SUMO-2 and SUMO-3 (Matic et al., 2008; 2010). However, the recent identification of non-canonical chains highlights the importance of exploring the potential for chain formation beyond the lysine residues within the consensus site (Gärtner et al., 2018). It is interesting to note that a group of enzymes called SUMO-targeted ubiquitin ligases (STUbLs) were discovered, which recognize SUMO polymers and ubiquitinate them, leading to the generation of hybrid chains. In mammals, two proteins belonging to this group have been identified to date: RING finger protein 4 (RNF4), which exhibits preference for SUMO-2 polymers (Tatham et al., 2008); and RING finger protein 111 (RNF111 or Arkadia), which ubiquitinates substrates carrying SUMO-2/3 polymers capped with SUMO-1 (Sriramachandran et al., 2019). Nonetheless, the available data regarding the function of mixed SUMO chains, the substrates that carry them and the structural arrangement of modified proteins remain quite constrained (examined in greater detail in Chang and Yeh, 2020b; Jansen and Vertegaal, 2021; Keiten-Schmitz et al., 2020).

SUMOylation is a process tightly linked to the regulation of protein-protein interactions. Consequently, numerous proteins possess SUMO-interacting motifs (SIMs) through which various protein complexes can be formed. Some of these domains exhibit distinct affinities for the three different paralogs of SUMO or even for certain SUMO chains. Even though this form of SUMO interaction is widely abundant, there are two other categories of SUMO interacting surfaces that, although not as thoroughly investigated, have been identified in a few proteins and can also contribute to the specificity towards a SUMO paralog (Brüninghoff et al., 2022). SUMO proteases have variable N-terminal regions that contain not only SIMs (Garvin et al., 2013; Wagner et al., 2019) but also other domains responsible for directing the deSUMOylase activity to distinct cellular compartments. Regulation and substrate selection of these enzymes can also be influenced by alternative splicing and/or post-translational modifications in these regions. Furthermore, the nine SUMO proteases described so far belong to three different families with different catalysis mechanisms. These mechanisms also play a role in determining the selectivity for particular SUMOylated substrates. In members of the SENP family, SUMO molecules enter the catalytic centre through a narrow tunnel covered with tryptophan residues that ensure the proper alignment of the diglycine motif and the scissile bond. These enzymes possess a notable characteristic: both the scissile bond and the isopeptide bond connecting the SUMO molecule and the substrate are positioned in a cis configuration, which is inherently unstable and thus, facilitates cleavage. SENP1 is essential for deSUMOylating modified substrates by SUMO-1 and processing SUMO precursors. SENP2 is mainly involved in deconjugating SUMO-2 from substrates. SENP3 and SENP5 exhibit a strong preference for processing and deconjugating SUMO-2 and SUMO-3. SENP6 and SENP7 primarily participate in the removal of SUMO moieties from substrates modified with polymeric chains of SUMO-2 and SUMO-3 (Nayak and Müller, 2014; Kunz et al., 2018; Chang and Yeh, 2020). The DESI family comprises two members, namely, DeSI-1 and DeSI-2, although only the former has been minimally characterized so far. DeSI-1 is able to deconjugate the three SUMO paralogs as well as SUMO-2 and SUMO-3 polymeric chains from zinc finger and BTB domain-containing protein 46 (BZEL), the sole substrate assigned to it until now. However, it exhibits a very low activity to process SUMO precursors. In order to carry out its catalytic activity, DeSI-1 forms a homodimer so that the two cysteine and histidine residues of the two subunits generate an active site in the groove between them (Shin et al., 2012; Suh et al., 2012). Lastly, a DUB belonging to the USP family, known as USPL1, has shown unique specificity for SUMO rather than ubiquitin. As a result, this protease features the typical right-hand scaffold of a USP catalytic domain but has replaced or deleted the essential residues for ubiquitin binding. Moreover, it also lacks the so-called blocking loops found in USPs. While USPL1 is capable of engaging with the three SUMO paralogs, it exhibits significantly greater activity towards SUMO-2 and SUMO-3. Nevertheless, the importance of its protease activity *in vivo* remains to be fully elucidated (Li et al., 2022).

Another UBL with the ability to form polymers is NEDD8. Although it has been possible for some time to generate poly-NEDD8 chains *in vitro* (Ohki et al., 2009), the occurrence of such chains *in vivo* has only recently been demonstrated (Vogl et al., 2020; Lobato-Gil et al., 2021). Furthermore, NEDD8 has also been found to form hybrid chains not only with ubiquitin but also with SUMO-2 (Vogl et al., 2020; Lobato-Gil et al., 2021). However, despite the recent findings, the biological implications of these chains at the cellular level as well as the E3 ligases and proteases involved in the generation and degradation of these chains have yet to be identified (more details in Meszka et al., 2022; Vijayasimha and Dolan, 2021). Despite the recognition of NEDD8 by certain DUBs like USP21 and UCHL3 as well as by numerous UBDs due to the high similarity between ubiquitin and NEDD8, this molecule also possesses a dedicated protease and distinct binding domain (Castagnoli et al., 2019). Although SENP8, also known as NEDP1 or Den1, belongs to the SUMO-specific protease family, it does not cleave SUMO but instead displays a great affinity for processing and cleaving NEDD8 molecules. While there is not unanimous consensus on the precise mechanism by which this protease can selectively identify NEDD8 over ubiquitin, it seems that specific amino acids in both molecules While there is not unanimous consensus on the precise mechanism by which this protease can selectively identify NEDD8 over ubiquitin, it seems that specific amino acids in both molecules may be critical in this process (Reverter et al., 2005; Shen et al., 2005; Shin et al., 2011).

Unlike other UBLs, UFM1 possesses a single C-terminal glycine positioned near a valine residue, which effectively blocks the recognition of UFM1 molecules by DUBs. UFM1 forms polymeric chains through its lysine 69 (Yoo et al., 2014; Peter et al., 2022) that are susceptible to cleavage by its two dedicated proteases: UFM1-specific protease 1 (UFSP1) and 2 (UFSP2). While the catalytic domains of these enzymes share structural similarities, alterations in three flexible regulatory loops impact the substrate specificity of the enzymes. Furthermore, UFSP2 bears an extra N-terminal domain critical for both substrate identification and localization to the endoplasmic reticulum, where it interacts with odorant response abnormal protein 4 (ODR4), which could allosterically regulate the catalytic activity of UFSP2. In contrast, UFSP1 is predominantly located in the cytosol (Millrine et al., 2023).

To date, there is no evidence to suggest that ISG15 can form homo-polymeric chains. However, Okumura et al. demonstrated that this unique UBL, consisting of two ubiquitin-like domains, has the capacity to form dimers via disulfide bonds through the cysteine 78 in the hinge region. This process is concentration dependent and reduces the availability of ISG15 monomers that can be conjugated to proteins by ISGylation (Okumura et al., 2008). The existence of dimers and even multimers has been supported by other studies but their cellular functions are not yet known (Sorensen et al., 2007; Napolitano et al., 2018). In addition, it has also been shown that ISG15 can be conjugated to lysines 29 and 48 of ubiquitin molecules, generating hybrid chains in fractions of certain cellular proteins. Hence, it seems that ISG15 might slow down the turnover of target proteins (Fan et al., 2015) or even inhibit mitophagy in Ataxia Telangiectasia cells (Juncker et al., 2021). However, it is not yet known how and which E3 ligases recognize these polyubiquitinated substrates and introduce ISG15 molecules to them. While various research studies have demonstrated that certain DUBs exhibit reactivity with ISG15, knock-in mice expressing catalytic inactive USP18 display elevated ISGylation levels. This observation implies that USP18 serves as the predominant deISGylase *in vivo* (Ketscher et al., 2015). Even though USP18 is a member of the USP family of DUBs, this protease does not exhibit any cross-reactivity with ubiquitin in mammals. This specificity is due to the ISG15-binding box 1 (IBB-1) and 2 (IBB-2) of USP18, which recognize regions of the C-terminal ubiquitin-like domain of ISG15 that are not found in ubiquitin. Analysis have revealed that IBB-1 plays the primary role in determining ISG15 specificity with IBB-2 making a minor contribution (Basters et al., 2017).

## 3 Non-catalytic functions of DUBs and UBL proteases

**FIGURE 1:**
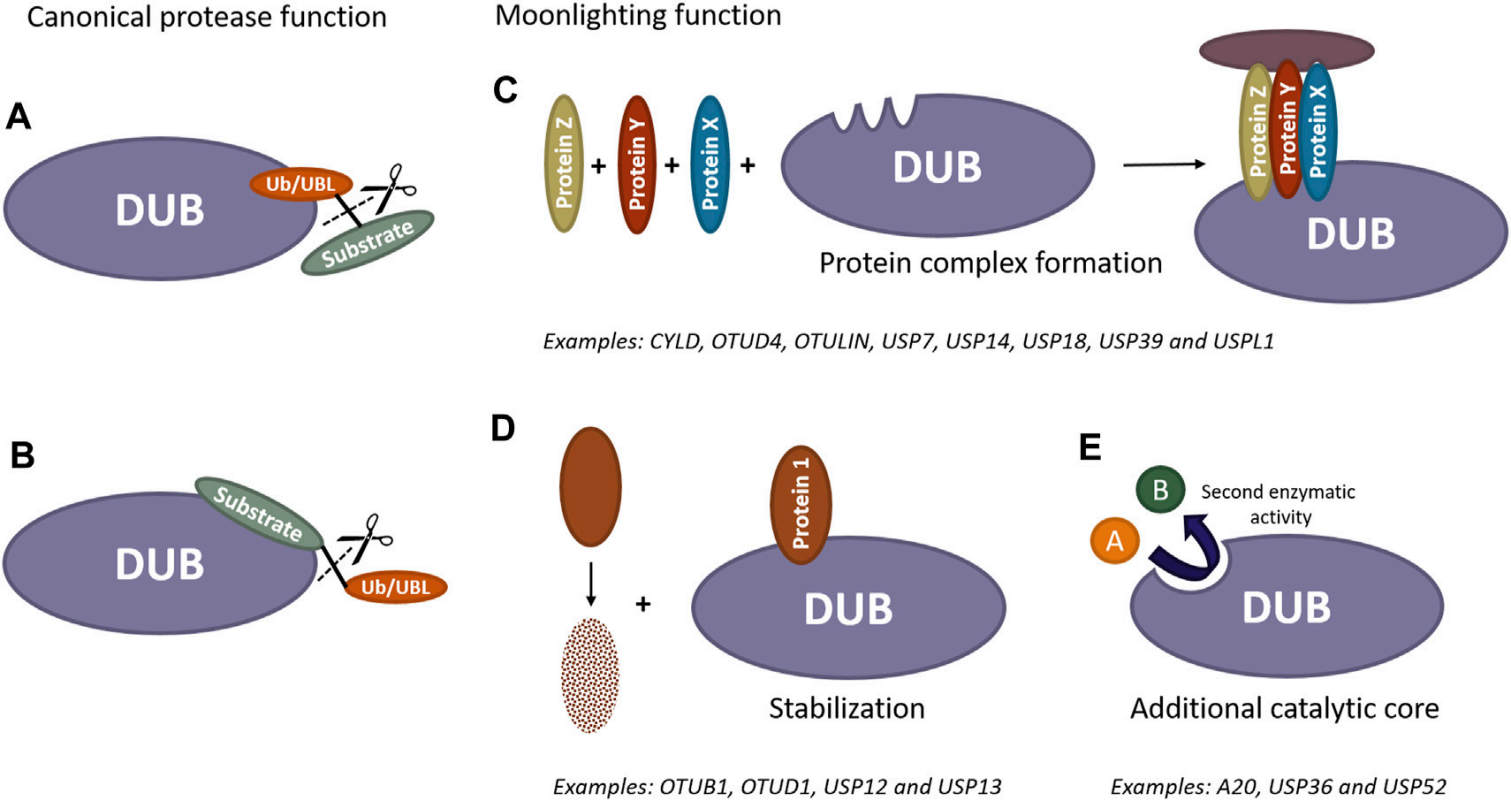
Scheme of catalytic and non-catalytic (moonlighting) functions of DUBs and UBL proteases. Canonically, these enzymes can remove ubiquitin/UBL molecules from modified substrates by recognizing either ubiquitin/UBL **(A)** or specific substrates **(B)**. Furthermore, these enzymes can exhibit additional activities independent of their catalytic function. DUBs and UBL proteases have the ability to recruit proteins and act as scaffold, forming new protein complexes **(C)**. They can also bind to proteins prone to degradation to prevent their destabilization **(D)**. Additionally, these proteases may contain extra catalytic cores, enabling them to perform entirely different functions beyond the removal of ubiquitin/UBL molecules from substrates **(E)**.

Over the last years it has been demonstrated that several DUBs and UBL proteases play essential roles in the organism that extend beyond their catalytic functions (Figure 1). Although these activities have only been investigated in depth in a handful of enzymes, more and more studies involving catalytically inactive mutants are being carried out to elucidate whether effects being observed after deletion of a particular protease are indeed caused by the lack of isopeptidase activity and not due to other functions unrelated to the catalytic function. Given the extensive repertoire of known DUBs and UBL proteases across various species, the available information on additional functions is minuscule and complicated by the fact that most of these proteases are characterized by a multi-domain architecture. This review aims to not only highlight the existence of these non-catalytic, moonlighting activities but also shed light on their functional and physiological importance. To this end, we have compiled and spotlighted those DUBs and UBL proteases that are already known to carry out activities beyond catalysis.

### 3.1 A20

Nuclear factor κB (NF-κB) is a family of inducible transcription factors involved in numerous cellular processes such as innate and adaptive immune responses, inflammation and cell adhesion and survival. These transcription factors become active in response to a diverse array of triggers including viral and bacterial pathogens, proinflammatory cytokines such as tumour necrosis factor (TNF) and inteleukin-1 (IL-1), genotoxic agents, ultraviolet radiation and oxidative stress. Under homeostatic conditions, NF-κB proteins form complexes with members of the inhibitory kB (IκB) family to hinder their translocation into the nucleus. In general, NF-κB activation takes place via both canonical and non-canonical pathways, each with specific functions. In the former one, proinflammatory cytokines and pathogen-associated molecular patterns (PAMPs) promptly induce the activation of the IKK complex, which consists of two kinase subunits, IKKα and IKKβ, as well as a regulatory subunit known as NEMO. Following activation, the IκB protein bound to the NF-κ dimer undergoes phosphorylation by IKK, inducing its polyubiquitination and ultimately its proteasomal degradation. Consequently, released NF-κB dimers migrate to the nucleus, where they induce the transcription of numerous genes. This transcriptional activity must be precisely regulated to prevent prolonged NF-κB activation, which could lead to adverse effects on the organism (Hayden and Ghosh, 2004; Tokunaga et al., 2012; Shi and Sun, 2018; Zhang et al., 2021).

One of the main negative regulators of NF-κB signalling, is A20, also known as TNFAIP3, whose expression is actually induced by the binding of NF-κB to two separated κB elements in its promoter (Krikos et al., 1992). A20 has been described as a ubiquitin-editing enzyme due to its ability to terminate NF-κB signalling by modifying the ubiquitination status of multiple proteins involved in this pathway. This protein contains a N-terminal OTU domain exhibiting DUB activity and seven zinc-finger (ZnF) domains that function as UBDs. So far, mainly the fourth and the seventh ZnF have been extensively studied. ZnF4 recognizes K63 polyubiquitin chains and functions as a ubiquitin E3 ligase (Bosanac et al., 2010), while ZnF7 shows high-affinity binding to M1-linked polymers (Tokunaga et al., 2012; Verhelst et al., 2012).

Undoubtedly, the function of A20 in the organism is crucial, as evidenced by the fact that knock-out mice of this protein die shortly after birth due to severe inflammation and cachexia (Lee et al., 2000). In humans, reduced expression of A20 has also been strongly associated with inflammatory and autoimmune diseases (Razani et al., 2020). Given that A20 is a DUB, it was initially believed and appeared a straightforward explanation that the catalytic function of this enzyme is critical for its essential function. However, meanwhile accumulated evidence suggests that the DUB activity of A20 might not be so relevant for the main physiological functions *in vivo*. Indeed, mice harbouring a point mutation in the catalytic cysteine of the OTU domain, which inactivates its protease activity, are healthy and do not exhibit the severe phenotype observed in A20 deficient mice (Lu et al., 2013; De et al., 2014; Wertz et al., 2015). Along the same lines, knock-in mice bearing a non-functional ZF4 domain are also grossly normal and do not exhibit an inflammatory phenotype (Lu et al., 2013; Wertz et al., 2015). In contrast, transgenic mice carrying a mutation in the ZnF7 domain that abrogates the binding to M1-linked chains have reduced body weight, splenomegaly and develop spontaneous inflammatory arthritis, suggesting that ZnF7 has a significant role in inhibiting inflammation *in vivo*, while the isopeptidase activity appears of minor importance (Polykratis et al., 2019). Nevertheless, the phenotype of these mice is still less severe than the one seen in A20 full knock-out mice, indicating that additional domains might also critically contribute to the negative regulation of NF-κB signaling by A20. Investigating the mechanisms of action of the remaining ZnF domains as well as inactivating multiple domains of A20 simultaneously will help to clarify their collaborative roles.

### 3.2 CYLD

Another major suppressor of NF-κB signalling is CYLD, which plays a regulatory function in a variety of cellular processes, including proliferation, survival and inflammatory responses. Mechanistically, CYLD possesses three cytoskeleton-associated protein-glycine-rich (CAP-Gly) domains at its N-terminal region, followed by a USP catalytic domain. In addition, CYLD contains two conserved proline-rich motifs and a TNF receptor associated factor 2 (TRAF2)-binding motif. Notably, the *CYLD* gene is of significant importance, as the loss of both alleles leads to the development of multiple benign skin tumours, referred to as cylindromatosis (Lork et al., 2017).

Aside from its DUB activity, CYLD also exerts non-catalytic functions through its CAP-Gly motifs. Specifically, the first two domains not only inhibit tubulin deacetylation by binding to the catalytic region of histone deacetylase 6 (HDAC6) but also contribute to the regulation of the perinuclear localization of CYLD and inducing a delay in cytokinesis (Wickström et al., 2010). Furthermore, the third CAP-Gly motif has been demonstrated to hinder Aurora kinase B activity by enhancing its dephosphorylation through protein phosphatase 2A (PP2A) recruitment. Although this function is independent of the DUB activity, the presence of the USP domain is still required (Sun et al., 2010). However, it is important to note that the CAP-Gly motifs, especially the third one, have been shown to enhance CYLD activity by predominantly boosting the affinity of the enzyme for ubiquitin chains. Moreover, these domains also contribute to the specificity of CYLD to cleave K63-linked ubiquitin chains. Nevertheless, it seems that the ubiquitin binding interfaces in each domain do not coincide with the established binding sites for tubulin or NEMO. This suggests that these interactions may either coexist or potentially collaborate, adding complexity to the analysis of the catalytic and non-catalytic functions of CYLD (Elliott et al., 2021).

CYLD deubiquitinates various substrates involved in NF-κB signalling, including NEMO. Originally, it was established that for this function, the third CAP-Gly motif of CYLD is needed to bind the zinc finger domain of NEMO (Saito et al., 2004). To further investigate the impact of CYLD-NEMO interactions, Zhao et al. generated a double mutant mouse model. In this model, NEMO carried a crucial mutation in its zinc finger domain and CYLD was knocked out, resulting in embryonic lethality. Mouse embryonic fibroblasts (MEFs) derived from these mice revealed that this mutant NEMO can no longer be recruited to the TNF receptor 1 (TNFR1) complex. As a consequence, cell death upon NF-κB induction by TNF occurs. Remarkably, the reconstitution of these MEFs with a catalytically inactive CYLD mutant could rescue the observed effects of TNF signalling, unequivocally showing that a non-catalytic function of CYLD, namely, acting as an adapter protein between TRAF2 and the NEMO zinc finger domain, is crucial during embryogenesis (Zhao et al., 2015a). Again, these results clearly show that effects from complete DUB deletion approaches need to be interpretated with caution and that in depth analysis is needed to unveil unexpected moonlighting functions of high physiological relevance.

### 3.3 OTUB1

Otubain-1 (OTUB1), one of the most highly expressed DUBs in humans and mice, is essential for embryonic development and homeostasis. Indeed, the vast majority of homozygous OTUB1 knock-out mice do not survive beyond the late stages of embryogenesis primarily due to lung inflation failure. Data suggest that this is a result of an increased proliferation of parenchymal and mesenchymal cells which leads to a reduction in saccular air space, potentially elevating lung tissue resistant to mechanical stretching (Ruiz-Serrano et al., 2021). Furthermore, it has also been demonstrated that OTUB1 deficient mice exhibit a significant delay in bone formation in the skull and limbs at embryonic day E16.5 when compared to control littermates, which becomes even more pronounced at E18.5 (Zhu et al., 2023). In adult mice, deletion of *Otub1* also induces proliferation in lung tissue and alveolar hyperventilation. Additionally, these mice are unable to effectively regulate and adjust their respiration in response to hypoxic conditions (Ruiz-Serrano et al., 2021). In a separate study, it was also demonstrated that the absence of Otub1 in adult mice enhances energy expenditure, decreases age-related body weight gain, improves blood glucose clearance and reduces baseline plasma insulin levels (Ruiz-serrano et al., 2022). However, the precise molecular mechanisms by which OTUB1 shapes these phenotypes are still not fully understood.

*In vitro* studies have shown that, at the molecular level, OTUB1 plays a pivotal role in regulating a multitude of cellular processes through its dual activity. On the one hand, OTUB1 is able to specifically remove K48 chains from different targets, preventing their degradation by the proteasome and, thus, increasing their half-life (Liu et al., 2022; Deng et al., 2023). This process involves the binding of the distal ubiquitin to the catalytic site of OTUB1, triggering a conformational change that enables the binding of the proximal ubiquitin to the N-terminal region of OTUB1, along with the proper positioning of the K48 scissile bond for cleavage (Wang et al., 2009). Remarkably, apart from its conventional DUB activity, OTUB1 can also modulate the ubiquitination status of several proteins by inhibiting the synthesis of K63 polyubiquitin chains that is performed by a specific subset of E2 enzymes in a non-catalytic manner. Intriguingly, OTUB1 carries out this function by creating a complex in which two ubiquitin molecules are arranged in a manner that resembles a cleaved K48-linked di-ubiquitin chain. OTUB1 binds to ubiquitin-charged E2 enzymes and positions the donor ubiquitin at its proximal site, thereby providing partial protection to the vulnerable thioester linkage through a helix situated in its N-terminal region. This process is allosterically regulated by free ubiquitin, which binds to the distal site of OTUB1 and enhances its affinity for E2 enzymes that are covalently linked to ubiquitin. Hence, OTUB1 hinders the formation of K63 polyubiquitin chains not only by interrupting the bond formation between donor ubiquitin and E2 enzyme but also by occluding their interaction with other proteins such as ubiquitin E2 variants (UEVs) and E3 ligases (Nakada et al., 2010; Juang et al., 2012; Wiener et al., 2012). Furthermore, one study has also revealed that OTUB1 can associate with ubiquitin conjugating enzyme E2 E1 (UBE2E1) through its non-catalytic activity, suppressing its autoubiquitination and hence, its proteasomal degradation. Given that many E2 enzymes of the UBE2E family undergo autoubiquitination, it is likely that OTUB1 is also involved in the regulation of their stability (Pasupala et al., 2018). Remarkably, the binding of uncharged E2 enzymes to OTUB1 stimulates its DUB activity (Wiener et al., 2013) showing that catalytic and non-catalytic functions can be intertwined.

Functionally, the non-catalytic function of OTUB1 is particularly important for the regulation of the DNA damage response, as it inhibits chromatin ubiquitination at DNA double-strand breaks, thereby preventing the recruitment of critical proteins for DNA repair (Nakada et al., 2010). OTUB1 also contributes to the regulation of apoptosis and transforming growth factor-β (TGFβ) signalling by hindering ubiquitination of p53 (Sun et al., 2012) and phospho-suppressor of mothers against decapentaplegic 2 and 3 (SMAD2/3) (Herhaus et al., 2013), respectively. Additionally, OTUB1 triggers the activation of the mitogen-activated protein kinase (MAPK) pathway by inhibiting RAS ubiquitination, promoting tumorigenic transformation (Baietti et al., 2016).

While both the catalytic and non-catalytic functions of OTUB1 are involved in numerous cellular processes, we presently lack an understanding of their specific physiological effects in the organism. It remains uncertain whether lack of both of these activities or just one of them is responsible for the lethality observed in OTUB1 deficient mice. It is therefore necessary to generate knock-in mice with targeted mutations to determine which of these functions is essential during embryogenesis.

### 3.4 OTUD1

Ubiquitination of serine/threonine kinase 1 (AKT1) with K63-linked chains is crucial for its recruitment to the plasma membrane, where it is phosphorylated and activated (Yang et al., 2009). Given that excessive activation of AKT1 is a prevalent factor in tumorigenesis, understanding the regulation of AKT1 is important to identify novel therapeutic targets in cancer. Unexpectedly, Fan et al. discovered that although OTUD1 is able to remove K63-linked ubiquitin chains from substrates, it also has the ability, independently of its catalytic activity, to strongly inhibit the phosphorylation of AKT1 and, consequently, its activation. OTUD1 comprises an N-terminal intrinsically disordered region followed by an OTU domain and an ubiquitin-interacting motif. Co-immunoprecipitation studies have revealed that a 36 amino acid peptide fragment (OUN-36) situated in the N-terminal region is sufficient to inhibit AKT1 phosphorylation and activation. OUN-36 binds to the pleckstrin homology (PH) domain of AKT1, preventing its interaction with phosphatidylinositol (3,4,5)-trisphosphate (PIP3) and thus, its recruitment to the cell membrane. Furthermore, administration of a recombinant fusion protein containing the OUN-36 fragment in combination with other anticancer drugs demonstrated increased sensitivity of tumour cells to targeted therapy and chemotherapy. This suggests that OUN-36 may become a promising protein drug for cancer therapy (Fan et al., 2023).

### 3.5 OTUD4

Diverse studies have uncovered essential functions of OTUD4 in different cellular processes that are not reliant on its DUB activity. Initially, OTUD4 has been demonstrated to be crucial for resistance to alkylation damage by stabilizing alpha-ketoglutarate dependent dioxygenase homolog 2 (ALKBH2) and 3 (ALKBH3). To achieve this, the 181–550 region of OTUD4, which does not include the OTU catalytic domain, forms a complex with USP7 and USP9X. These two enzymes remove the K48-linked ubiquitin chains of ALKBH2 and ALKBH3 by their DUB activity, thereby preventing their degradation by the proteasome (Zhao et al., 2015b).

Despite lacking a conventional RNA-binding domain, OTUD4 has been found to interact with RNA as well as with other RNA-binding proteins. While the precise regions involved in these bindings have not yet been identified, OTUD4 has been shown to play a crucial role in the formation of stress granules. These membrane-less cellular compartments that contain numerous RNAs and associated proteins are generated in response to brief stress exposure. Remarkably, the function of OTUD4 associated with stress granule formation was shown to be entirely independent of its catalytic activity. The same study indicates that OTUD4 is also a component of neuronal RNA transport granules under physiological conditions. These granules facilitate the transport of mRNAs from the cell body to the axon and dendrites, enabling local translation. However, it remains uncertain whether OTUD4 exerts DUB activity in these neuronal RNA granules. Additionally, data suggest that OTUD4 may be involved in the regulation of translation, but further research is required to clarify the veracity of this hypothesis (Das et al., 2019) and its dependence on protease or non-protease activity.

OTUD4 has also been shown to play a non-catalytic role in regulating the TGFβ signalling, particularly under basal conditions. In the absence of ligand, OTUD4 is able to increase the levels of TGFβ receptor subunit I at the plasma membrane by maintaining SMAD specific E3 ubiquitin protein ligase 2 (SMURF2) in a closed, inactive and stable conformation. As a result, SMURF2 cannot ubiquitinate the TGFβ receptor complex, preventing its degradation. Nevertheless, the complete understanding of the molecular mechanisms by which OTUD4 performs this function, as well as the specific regions of the enzyme involved, are not yet fully understood (Jaynes et al., 2020).

Lastly, in more recent findings, it has been demonstrated that while the DUB activity of OTUD4 is crucial for the formation of processing bodies in neural progenitor cells during brain development, OTUD4 also regulates neuronal migration to the cortical plate through a non-catalytic mechanism (Kedia et al., 2022).

### 3.6 OTULIN

The majority of recognized functions of OTULIN are attributed to its role in ubiquitin homeostasis by removing Met1-linked ubiquitin chains from substrates and binding to the linear ubiquitin chain assembly complex (LUBAC). Although there is no doubt that the catalytic activity of OTULIN is essential for the organism, as evidenced by embryonic lethality in mice expressing a catalytic dead mutant of this enzyme (Heger et al., 2018); it is important to note that, irrespective of its protease activity, OTULIN also plays a vital role in regulating sorting nexin 27 (SNX27)-dependent cargo loading, retromer assembly and endosome-to-plasma membrane recycling. In order to perform these non-catalytic functions, OTULIN establishes a high-affinity interaction with the cargo binding pocket of SNX27 through two distinct surfaces: one involving a conserved PSD95–Dlg1–ZO-1 (PDZ)-binding motif at its C-terminal region and the other comprising some conserved residues in the OTU domain that are not involved in the catalytic activity of the enzyme. When bound to OTULIN, SNX27 fails to recruit the retromer subunits vacuolar protein sorting-associated proteins 26A (VPS26) and 35 (VPS35), hindering the formation of a functional SNX27-retromer complex and thus, inhibiting the recycling of plasma membrane receptors. Conversely, binding of OTULIN to SNX27 does not inhibit the DUB activity of OTULIN. The mechanisms by which the interaction between OTULIN and SNX27 breaks down and SNX27 can form the retromer are currently unknown. However, one study suggests that PTMs may be implicated. Furthermore, the physiological implications of the OTULIN-SNX27 interaction need to be investigated (Stangl et al., 2019).

### 3.7 USP7

USP7 is a large enzyme with a N-terminal tumour necrosis factor receptor-associated factor (TRAF) domain (NTD) followed by the USP domain and five successive UBL domains. The catalytic domain alone exhibits minimal activity but requires UBL domains four and five for full DUB activity. In contrast, the remaining UBL domains do not impact the catalytic activity but play a role in mediating specific interactions with various proteins (Rougé et al., 2016). Noteworthy is the direct interaction of USP7 with both p53 and murine double minute 2 (MDM2), highlighting its significant role in regulating growth arrest, DNA repair and apoptosis, particularly in cancer. Moreover, USP7 also modulates other cellular processes, including immune responses and viral replication (Harakandi et al., 2021).

The significance of USP7 is clearly evident as USP7 knock-out mice die during the early stages of embryonic development, between embryonic days E6.5 and E7.5, associated with profound p53 activation. Intriguingly, the lethality observed in these mice is not rescued in the absence of p53, indicating that USP7 may have p53-independent functions that are crucial for proliferation and differentiation (Kon et al., 2010). Indeed, it has been recently demonstrated that USP7 has a previously unknown function in maintaining the identity of mouse embryonic stell cells (mESCs) by repressing lineage differentiation genes. Remarkably, this is achieved through both catalytic and non-catalytic functions. On the one hand, USP7 regulates the ubiquitination status of histones and proteins involved in the transcriptional network of mESCs via its DUB activity. Particularly, USP7 deubiquitinates and stabilizes sex determining region Y-box 2 (SOX2), thereby repressing the transcription of mesoendodermal linage genes. On the other hand, USP7 is able to maintain RING1 and YY1 binding protein (RYBP) binding on chromatin independently of its catalytic activity. Consequently, expression of primitive endoderm-associated genes is hindered. Despite these findings, it remains to be established whether these newly identified functions of USP7 play a critical role in the early stages of embryonic development (Liu et al., 2023) and whether USP7 is able to modulate other cellular processes in a non-catalytic manner.

### 3.8 USP12

USP12 is a cysteine protease that is responsible for trimming polyubiquitin chains from various substrates such as histones H2A and H2B (Joo et al., 2011), the nonactivated form of Notch (Moretti et al., 2012), androgen receptor (Burska et al., 2013; McClurg et al., 2018), PH domain and leucine rich repeat protein phosphatase 1 (PHLPP1) (Gangula and Maddika, 2013) and MDM2 (McClurg et al., 2018). The DUB activity of USP12 can be stimulated through the interaction with WD repeat domain 20 (WDR20) and 48 (WDR48) (Burska et al., 2013; Gangula and Maddika, 2013). Although USP12 modulates various cellular processes, it has a notable impact on tumorigenesis, as evidenced by its overexpression in a wide range of cancers (Zhang et al., 2016; Niu et al., 2023).

Interestingly, USP12 has also been shown to possess non-catalytic functions, which are particularly significant in Huntington’s disease. This neurodegenerative condition arises from the abnormal expansion of CAG trinucleotide repeats in the huntingtin (*HTT*) gene, leading to the accumulation of toxic protein species. A study in yeast found that Ubp13, the yeast orthologue of USP12, enhances the toxicity of mutant HTT fragments (Willingham et al., 2003). Following studies by Aron et al. demonstrated that USP12 suppresses the toxicity of these mutated proteins by inducing autophagy in neurons. This function was shown to be independent of its catalytic activity in three different model systems: rat primary neurons, human patient-induced pluripotent stem cell-derived neurons and in a *Drosophila* model of Huntington’s disease. However, USP12 does not generically suppress neurotoxicity, as overexpression of this enzyme in neuronal models of Parkinson’s and Alzheimer’s disease does not rescue the neuronal toxicity induced by TAR DNA-binding protein 43 (TDP-43) and α-synuclein. While the specific molecular mechanisms underlying this activity of USP12 remain elusive, it has been shown that USP46 cannot compensate for this neuroprotective function despite sharing 93% protein sequence similarity and an identical catalytic domain with USP12 (Aron et al., 2018).

Additionally, another study has demonstrated that USP12 can inhibit CREB-binding protein (CBP)-mediated acetylation in cells in a non-catalytic manner. While interferon (IFN) stimulates the transport of CBP from the nucleus to the cytoplasm, it also induces the nuclear import of USP12. Thus, CBP can induce the acetylation of both interferon alpha/beta receptor chain 2 (IFNAR2) and signal transducer and activator of transcription 2 (STAT2), promoting the formation of the ISG factor 3 (ISGF3) complex and thereby enhancing the transcription of ISG genes. Conversely, in the nucleus, USP12 binds to the histone acetyltransferase (HAT) domain of CBP, inhibiting acetylation of phosphorylated STAT1 and thus, maintaining IFN signalling (Liu et al., 2020). Further investigations are required to elucidate how USP12 is translocated to the nucleus. Likewise physiological implications of this non-catalytic function of USP12 on the immune response remain to be investigated.

### 3.9 USP13

USP13, a member of the USP family, possesses a distinctive structure with a catalytic domain embedded with two ubiquitin-associated (UBA) domains and a ZnF motif at the N-terminal region that notably does not bind ubiquitin. Despite its relatively weak DUB activity *in vitro* and in cells, USP13 is implicated in regulating several crucial cellular processes by removing ubiquitin from various substrates (Li et al., 2022). For example, it modulates mitochondrial energy metabolism by detaching K48-linked ubiquitin chains from key proteins such as ATP citrate lyase (ACLY) and oxoglutarate dehydrogenase (OGDH) (Han et al., 2016). USP13 also promotes autophagy by deubiquitinating VPS34 (Xie et al., 2020). In the endoplasmic reticulum-associated protein degradation (ERAD) process, it collaborates with the E3 ubiquitin ligase glycoprotein 78 (Gp78) to maintain a balance between ubiquitination and deubiquitination (Liu et al., 2014). Furthermore, USP13 is involved in the DNA damage response by deubiquitinating proteins like receptor-associated protein 80 (RAP80) (Li et al., 2017), DNA topoisomerase II binding protein 1 (TOPBP1) (Kim et al., 2021) and high mobility group box 1 (HMGB1) (Shin et al., 2022). Intriguingly, it also influences the nuclear-to-cytoplasmic translocation of HMGB1, although the exact molecular details are yet to be elucidated (Shin et al., 2022). Overall, it is thought that the observed weak DUB activity of USP13 might be due to an autoinhibitory mechanism involving interactions between its UBA and ZnF domains, which is hypothesized to be reversed by protein recruitment and/or PTMs. Nonetheless, detailed investigations into this regulation and the validation of this proposition are still pending (Li et al., 2022).

Studies involving catalytically inactive mutants unequivocally indicate that the DUB activity of USP13 is vital for maintaining the homeostasis of many cellular processes. However, recent findings have uncovered additional roles for USP13 beyond its catalytic activity. Specifically, Esposito et al. found that Aurora kinase B, crucial for mitosis, phosphorylates USP13 at the serine 114 and that this phosphorylation enhances the interaction of both proteins, thereby maintaining the stability of Aurora kinase B. Intriguingly, USP13 stabilizes Aurora kinase B by modulating its ubiquitination, a process that occurs independently of its DUB activity. This is evidenced by the fact that the catalytically inactive C345A mutant of USP13 still stabilizes and deubiquitinates Aurora kinase B in cells. The exact mechanisms of this process remain unclear but it has been proposed that USP13 might either shield Aurora kinase B from being ubiquitinated or collaborate with other DUBs to deubiquitinate it (Esposito et al., 2020).

### 3.10 USP14/Ubp6

In eukaryotes, the ubiquitin-proteasome system (UPS) is responsible for selectively degrading and recycling most cellular proteins. Initially, polypeptides that need to be eliminated are tagged with polyubiquitin chains and then transported to the 26S proteasome, where they undergo deubiquitination, unfolding and degradation. Whereas the breakdown of the polypeptide occurs within the cylindrical 20S core particle of the 26S proteasome, deubiquitination and unfolding are performed by proteins located within the one or two 19S regulatory particles that cap the core particle. Within these regulatory particles are subunits Rpn1, Rpn10 and Rpn13, which act as ubiquitin receptors, as well as DUBs Rpn11, Uch37 and USP14, responsible for eliminating the polyubiquitin chains that are attached to the marked protein. Notably, Rpn11 is an integral subunit of the 19S regulatory particle whereas Uch37 and USP14 associate reversibly with the proteasome (Hung et al., 2022; Zhang et al., 2022).

When not associated with the proteasome, USP14 and its yeast orthologue Ubp6 are in an inactive state due to the presence of two blocking loops that obstruct the ubiquitin C-terminal binding groove. Once the N-terminal UBL domain of USP14 and Ubp6 binds to Rpn1, these blocking loops within the USP domain engage with both the oligonucleotide/oligosaccharide-binding (OB) and AAA domains of the adenosine triphosphatases (ATPases) ring of the regulatory particle. This increases the catalytic activity of USP14 and Ubp6 over 800- and 300-fold, respectively. Accordingly, USP14 and Ubp6 efficiently trim polyubiquitin chains attached to target proteins, thereby promoting the recycling of ubiquitin molecules. Irrespective of their isopeptidase functions, USP14 and Ubp6 are also major allosteric regulators of the proteasome. Both DUBs possess the remarkable ability to both stimulate and inhibit multiple steps during proteasomal degradation, independently of their catalytic activity. In the presence of substrate, USP14 and Ubp6 enhance the ATPase rate, strengthen the interaction between the regulatory and the core particles and induce early gate opening. Conversely, in the absence of substrate, binding of the UBL domain of USP14 or Ubp6 to the regulatory particle leads to the suppression of the proteasomal activity, delaying protein degradation. Although USP14 and Ubp6 perform this inhibitory function independently of their catalytic activity, it requires the presence of a ubiquitin molecule bound to their catalytic domain. Therefore, the ability of USP14 and Ubp6 to inhibit the proteasome in a non-catalytic manner relies on both UBL and USP domains of these proteins (Hanna et al., 2006; Kim and Goldberg, 2017; Hung et al., 2022; Zhang et al., 2022).

Dysfunction of the UPS has been associated with numerous diseases, particularly neurodegenerative pathologies such as Alzheimer’s or Parkinson’s disease (Thibaudeau et al., 2018). Ataxic mice develop a spontaneous mutation in *USP14* that results in a 95% reduction of the protein, leading to reduced muscle mass, rigidity, tremor and ultimately early perinatal lethality. These mice exhibit a neuromuscular phenotype with localized synaptic alterations at the neuromuscular junctions. However, in contrast to other neurodegenerative disorders such as Parkinson’s disease, the central nervous system of these mice does not exhibit detectable ubiquitin aggregates or loss of neuronal cells (Wilson et al., 2002). Reintroduction of *USP14* exclusively in the nervous system of ataxic mice is sufficient to correct all aspects of the neuromuscular phenotype, suggesting that USP14 plays a critical role in neuromuscular junctions’ development as well as in maintaining synaptic activity (Chen et al., 2009). Moreover, complementation of ataxic mice with neuronally expressed ubiquitin also corrects developmental and functional deficits caused by USP14 loss, indicating that the DUB activity of USP14 is crucial for preserving the pool of free ubiquitin in the nervous system for normal synaptic function (Chen et al., 2011). Conversely, a different study has demonstrated that in the hippocampus, the absence of USP14 leads to a reduction in both docked and total presynaptic vesicles. Of note, this effect can be reversed by introducing a catalytically inactive USP14 mutant or by inhibiting the proteasome, but not by ubiquitin expression. These findings clearly imply that USP14 regulates synaptic structure and short-term plasticity in a non-catalytic manner (Walters et al., 2014).

Studies in yeast have revealed that certain phenotypes resulting from the absence of Ubp6, such as increased sensitivity to canavanine or some translational inhibitors, can be rescued by overexpression of ubiquitin. However, other phenotypes, like the inhibition of the target of rapamycin (TOR) signalling pathway, which is responsible for regulating the overall condition of yeast cells in response to nutritional conditions, are not influenced by ubiquitin. Interestingly, expression of a catalytically inactive form of Ubp6 complemented rapamycin resistance, strongly suggesting that Ubp6 may possess additional non-catalytic functions (Hanna et al., 2006).

### 3.11 USP18

As previously mentioned, USP18 is a specific ISG15 protease and consequently, it lacks DUB activity but exhibits deISGylase activity instead. While USP18 knock-out in C57BL/6 mice causes embryonic lethality (Kim et al., 2005); it leads to a decreased life expectancy and a pronounced brain phenotype in a mixed 129_C57BL/6 genetic background. More recently, it was shown that USP18 knock-out mice exhibit significantly smaller fetal livers compared to their control littermates at embryonic day E14.5 (Arimoto et al., 2023). In humans, USP18 deficiency also results in early mortality during childhood and a remarkable brain phenotype akin to that observed in mice (Meuwissen et al., 2016). As it was detected that elimination of USP18 results in increased protein ISGylation levels in the brain, it was initially concluded that ependymal cell necrosis and hydrocephalus development is caused by this aberrant stabilization of ISG15 modification (Ritchie et al., 2002). However, investigations with double knock-out mice lacking both USP18 and ISG15 revealed an identical phenotype to that of USP18 deficient mice, clearly demonstrating that increased ISGylation levels cannot be the underlying cause of the observed brain phenotype (Knobeloch et al., 2005). In fact, reconstitution of USP18 deficient MEFs with a catalytically inactive enzyme also resulted in lower levels of ISG15 conjugates, suggesting that USP18 might have a distinct function unrelated to its catalytic activity (Malakhova et al., 2006).

The hypersensitivity to type I IFN treatment in both USP18 deficient mice as well as USP18 and ISG15 deficient mice led to the hypothesis that USP18 might function as a negative regulator of IFN signalling (Malakhova et al., 2003; Knobeloch et al., 2005). Subsequent investigations not only confirmed the ability of USP18 to terminate the IFN signalling pathway but also revealed that this regulatory function is independent of its catalytic activity (Malakhova et al., 2006). Concordantly, knock-in mice expressing catalytically inactive USP18 (USP18^C61A/C61A^) demonstrate a normal lifespan, absence of brain abnormalities and exhibit no hypersensitivity to IFN. These results provided clear evidence that the function of USP18 as a major negative regulator of type I IFN signalling is entirely unrelated to its protease activity. Nevertheless, as expected, USP18^C61A/C61A^ mice display increased levels of ISG15 conjugates, further confirming that USP18 is the main ISG15 isopeptidase *in vivo* (Ketscher et al., 2015). Although the precise molecular mechanisms underlying the non-catalytic function of USP18 remain incompletely understood, data suggest that there is a direct interaction between USP18 and STAT2, which is crucial for recruiting USP18 to the plasma membrane, where it competes with janus kinase 1 (JAK1) for binding to IFNAR2. Thus, there is a reduction in ternary-complex formation as well as signal activation at the plasma membrane. In line with this mechanism, USP18 cannot fulfil its role as a negative regulator of IFN signalling in the absence of STAT2 (Malakhova et al., 2006; Arimoto et al., 2017). Likewise, humans expressing STAT2 mutant proteins unable to interact with USP18 exhibit uncontrolled IFN activation and develop fatal interferonopathies (Duncan et al., 2019; Gruber et al., 2020; Martín-Fernandez et al., 2022).

Remarkably, it has been demonstrated that, at least in humans, unconjugated ISG15 also plays a pivotal role in negatively regulating the IFN signalling pathway by preventing the proteasomal degradation of USP18. Intriguingly, this mechanism is not observed in mice, where USP18 does not require ISG15 binding to terminate the IFN signalling (Speer et al., 2016). However, the precise mechanisms remain unknown. Additionally, it is unclear whether free ISG15 is recruited to the plasma membrane alongside USP18 or how STAT2 is precisely involved.

Recently, an additional non-catalytic role has been ascribed to USP18. Arimoto et al. have discovered that USP18 can also interact with STAT2 in the nucleus, inhibiting the binding of ISGF3 complexes to IFN-stimulated response (ISRE) DNA elements and restricting the transcription of ISGs genes. Surprisingly, USP18 also exerts control over the expression of genes implicated in inflammatory responses by impeding the interaction of the complex formed by STAT2, IFN regulatory factor 9 (IRF9) and p65 with NF-κB motifs in the promoter/enhancer regions of NF-κB-related regulators. Among these genes are those associated with nucleotide-binding oligomerization domain-like receptor protein 3 (NLRP3) inflammasome activation and polo-like kinase 2 (PLK2), unveiling a novel function of USP18 as a safeguard for malignant cells against IFN-induced pyroptosis. Notably, this protective function appears to be independent of the catalytic activity of USP18 but relies on its interaction with STAT2 (Arimoto et al., 2023).

Different studies have demonstrated that USP18 is notably overexpressed in a variety of cancer types. Both the catalytic and non-catalytic functions of this protease play a role in tumourigenesis. In most cases, suppressing USP18 has been found to decrease the stability of key growth-regulatory molecules, leading to reduced cancer cell proliferation and tumour growth. This suppression has been shown to enhance apoptosis and sensitize cancer cells to chemotherapeutic treatments, thereby hindering cancer progression and improving survival rates (Honke et al., 2016; Mustachio et al., 2018). Notably, a recent study using established murine leukemia models have revealed that even a partial reduction of USP18 is sufficient to produce these anti-tumour effects without adversely impacting normal cells. Furthermore, USP18 inhibition specifically targets cancer stem cells, which are crucial for cancer recurrence (Arimoto et al., 2023). A recent study has introduced the idea that inhibiting USP18 in macrophages could reprogram tumour-associated macrophages to boost their anti-tumour functions in various cancer types. This study, led by Miyauchi et al., found that the reduction of USP18 enhances the proteasomal degradation of colony stimulating factor 1 receptor (CSF1R) mediated by ubiquitin conjugating enzyme E2 D1 (UBCH5) and neural precursor cell expressed developmentally downregulated 4 E3 ubiquitin protein ligase (NEDD4). CSF1R plays a pivotal role in driving macrophages towards a tumour-promoting phenotype. Consequently, diminishing USP18 leads to an increase in anti-tumour macrophages within the tumour microenvironment. Given that myeloid cells often make up 30%–50% of the immune cells infiltrating tumours, targeting these cells could be a more viable and effective approach compared to current therapies focused on tumour-infiltrating T cells, which are present in significantly lower numbers. Therefore, the strategy of targeting USP18 in myeloid cells holds substantial promise as a therapeutic approach in a variety of cancer types (Miyauchi et al., 2023). Nonetheless, it must not be overlooked that in certain types of cancer such as melanoma or leiomyosarcoma, USP18 expression is beneficial for anti-cancer immunity and tumour growth suppression. Consequently, the specific impact of USP18 inhibition needs to be carefully assessed for each type of cancer (Honke et al., 2016; Mustachio et al., 2018).

### 3.12 USP36

USP36 stands out as a unique and intriguing protein due to its exclusive localization in human nucleoli, where it plays a dual and crucial role in ribosome biogenesis, a highly coordinated cellular process involving the synthesis and processing of ribosomal RNA, the assembly of ribosome subunits in the nucleolus and their eventual transport into the cytoplasm. On the one side, USP36 deubiquitinates several proteins such as RNA polymerase I subunit A, DEAH-box RNA helicase DHX33, nucleophosmin and, notably, the proto-oncogenic regulator Myc (van den Heuvel et al., 2021). On the other side, it acts like a SUMO ligase to directly mediate the SUMOylation of nucleolar proteins, especially those associated with ribosome biogenesis. Ryu et al. demonstrated that USP36 associates with the two major classes of small nucleolar ribonucleoproteins (snoRNPs), box C/D and box H/ACA, which are responsible for 2′-O-methylation and pseudouridylation of specific nucleotides, respectively. They found that USP36 SUMOylates two proteins in each of these complexes: Nop58 and Nop56 in box C/D and Nhp2 and DKC1 in box H/ACA (Ryu et al., 2021). Moreover, Chen et al have shown that USP36 also mediates the SUMOylation of exosome component 10 (EXOSC10), critical for the RNA exosome function in ribosome biogenesis (Chen et al., 2023).

Interestingly, both DUB and SUMOylase activities of USP36 are localized within its N-terminal USP domain. However, they are independent of each other, as evidenced by the H382 mutant which, despite lacking DUB function, retains its ability to promote SUMOylation. The loss of the SUMOylase activity in the catalytically inactive mutant C131 suggests that the conserved cysteine may play a structural role for SUMOylation. Nevertheless, further research is required to unravel the molecular mechanisms underlying the interplay between DUB and SUMO E3 ligase activities of USP36 as well as to discover mutants that selectively disable the SUMOylase function (Ryu et al., 2021).

In addition to all this, it is worth noting that recent discoveries have highlighted the versatility of USP36 as a cross-reactive DUB, capable of processing not only ubiquitin but also FUBI (van den Heuvel et al., 2021; O’Dea et al., 2023) and ISG15 (Zhao et al., 2023).

### 3.13 USP39/Sad1

Despite having a USP domain, USP39 lacks the conserved catalytic histidine and cysteine residues typically required for DUB activity. Additionally, its N-terminal ZnF domain is incapable of binding ubiquitin. Therefore, it was originally thought that USP39, like its yeast orthologue Sad1, are deprived of this catalytic function (Van Leuken et al., 2008; Hadjivassiliou et al., 2014). However, recent studies have introduced the intriguing possibility that USP39 and Sad1 might retain the ability to detach ubiquitin chains from substrates, even though the conventional cysteine residue in their catalytic triads is replaced with an aspartic acid. Chen et al. recently reported that USP39 enhances SARS-CoV-2 replication by deubiquitinating and stabilizing the envelope protein of this virus. Their findings reveal that USP39 engages with this protein through the arginine-rich motif in its N-terminal region and carries out its DUB activity via the USP domain. Mutations in either domain were found to disrupt the stabilization of the viral protein. Furthermore, they demonstrated that despite USP14 being co-pulled down with the envelope protein, this protease did not play a role in its deubiquitination (Chen et al., 2024). Another study revealed that USP39 has also a direct DUB activity towards the regulation of zinc-finger E-box-binding homeobox 1 (ZEB1), a key promoter of epithelial-to-mesenchymal transition that enhances tumour growth and metastasis. Transfecting the USP domain of the protease into USP39 knockdown cells effectively reduced the ubiquitination level of ZEB1 (Li et al., 2021). Considering USP52 as a benchmark (discussed below), which also lacks the standard catalytic triad yet exhibits DUB activity though non-conserved cysteine residues (Yang et al., 2018), it is essential to thoroughly examine the cysteines in USP39 to conclusively determine its DUB potential and the mechanisms behind it. Various studies have already begun this exploration: some propose that the cysteine at position 306 might contribute to the catalytic function (Wu et al., 2019; Dong et al., 2021), while another suggests the involvement of cysteines 136 and 139 (Peng et al., 2020), despite their location outside the USP domain. A meticulous investigation of the amino acid residues, along with structural data, is indeed crucial to reach a consensus on the catalytic capabilities of USP39.

Another factor advocating for a re-examination of the DUB activity of USP39 and Sad1 is the recent discovery that mice lacking USP39 exhibit embryonic lethality, partly due to abnormal mesoderm migration. Interestingly, this phenotype can be partially rescued by deleting one allele of the ubiquitin like modifier activating enzyme 1 (*Ube1*) gene, which leads to a decrease in the global ubiquitination level (Kimura-Yoshida et al., 2022). Additionally, research in yeast has demonstrated that Sad1 is crucial for cell viability, with both its ZnF domain and USP domain being essential for it (Huang et al., 2014).

Regardless of their putative DUB activity, USP39 and Sad1 are essential in the process of pre-messenger RNA (pre-mRNA) splicing. They facilitate the integration of the triple small nuclear ribonucleoprotein (tri-snRNP) into the pre-spliceosome, which is a crucial step in forming the mature spliceosome (Makarova, 2001; Hadjivassiliou et al., 2014). However, the precise molecular mechanisms by which they manage this recruitment are not yet fully understood. Nevertheless, USP39 is indispensable for the splicing of a wide array of mRNAs, including those for Aurora kinase B (Van Leuken et al., 2008) and various autophagy-related genes (Cui et al., 2023), thereby playing a significant role in a multitude of cellular processes. Furthermore, it has been demonstrated that USP39 specifically influences immunoglobin gene rearrangement through a mechanism that depends on the spliceosome but is independent of RNA splicing. This effect is likely achieved by altering chromatin interactions at the immunoglobulin heavy (*Igh*) locus, although the exact mechanisms behind this are still under investigation (Ruan et al., 2022).

Another study has revealed that USP39 rapidly localizes to DNA lesions, attaching onto poly (ADP-ribose) (PAR) chains through its tripartite glycine-arginine-rich motif situated in the N-terminal region, a feature unique to mammals, thus promoting liquid demixing. Furthermore, USP39, through mechanisms that overlap with its splicing function, uses its ZnF domain to assist in assembling non-homologous end-joining (NHEJ) factors, while employing its USP domain to regulate the process of homologous recombination repair (Kim et al., 2021).

### 3.14 USP52

Initially considered inactive (Walden et al., 2018), USP52, also referred to as poly(A) specific ribonuclease subunit 2 (PAN2), was found by Yang et al. to exhibit DUB activity (Yang et al., 2018). Although the exact residues responsible for this catalytic function remain unidentified, they hypothesized that specific PTMs or protein folding might rearrange other cysteine and histidine residues present in the protease to create a new active centre capable of hydrolysing ubiquitin chains. Consequently, their research demonstrated that USP52 facilitates anti-silencing function 1A histone chaperone (ASF1A) deubiquitination and stabilisation, thereby supporting chromatin assembly and promoting cell cycle progression (Yang et al., 2018).

In eukaryotic cells, mature mRNAs transported from the nucleus to the cytoplasm typically feature a 3’ poly(A) tail and a 5’ 7-methylguanylate (m7G) cap, with the exception of histone coding mRNAs that lack the poly(A) tail. These structural features not only safeguard mRNA from nonspecific degradation by 3’-5’ exoribonucleases but are also essential for efficient translation (Zhao et al., 2023). Beyond its recently identified DUB activity, USP52 or PAN2 plays a crucial role in the poly(A) tail shortening in mRNA, a process termed deadenylation. This process is the first rate-limiting step in eukaryotic cells and is closely. linked with translation, significantly influencing gene expression regulation. To perform this function, PAN2 associates with a homodimer of the regulatory subunit 3 (PAN3), creating a PAN nuclease complex. PAN2 serves as the catalytic component of this complex, possessing intrinsic 3’ to 5’ exoribonuclase activity, which is attributed to its C-terminal aspartate-glutamate-aspartate-aspartate (DEDD) domain. Notably, PAN2 alone demonstrates limited affinity for RNA and exhibits relatively modest catalytic efficiency without the presence of PAN3. Instead, PAN2 exhibits significant deadenylation activity and a preference for poly(A) once it binds to PAN3 (Uchida et al., 2004; Zhao et al., 2023).

### 3.15 USPL1

As outlined above, USPL1 is a SUMO protease that neither binds nor cleaves ubiquitin. Beyond its deSUMOylase activity, USPL1 has been found to exert essential non-catalytic functions. USPL1 is localized in Cajal bodies, nuclear organelles that undergo changes in number, size and composition during cell cycle, development and stress. These coiled bodies carry out various functions, including telomere maintenance, histone messenger RNA processing and maturation and assembly of small nuclear ribonucleoprotein particles. Studies have revealed that the knock-out of USPL1 in cells not only alters the number and size of Cajal bodies but also halts cell proliferation. The fact that transfection with a catalytically inactive mutant can rescue the observed effects suggests that USPL1 performs this function independently of its deSUMOylase activity (Schulz et al., 2012). Further studies have revealed that USPL1 interacts with components of the little elongation complex (LEC) and is present at snRNA gene loci in human cells, suggesting that USPL1 may have an important role in small nuclear ribonucleoproteins (snRNP) biogenesis at the transcriptional level (Hutten et al., 2014).

The importance of USPL1 has been emphasized in zebrafish, where the inactivation of c13orf22l, the zebrafish homolog of USPL1, results in embryonic lethality marked by a distinct necrotic phenotype in the central nervous system and eyes within 2 days post-fertilization. Examination of head regions at this developmental stage further unveiled a significant accumulation of coilin in the nucleoli (Amsterdam et al., 2004; Schulz et al., 2012). Further studies in mammals will help to better understand the function of USPL1, which has so far been poorly studied.

## 4 Pseudo-DUBs

Approximately 13% of the DUBs identified to date are considered catalytically inactive, known as pseudo-DUBs, due to their lack of the necessary catalytic residues for ubiquitin cleavage (Chen et al., 2024). The distribution of these proteins varies significantly across different families. For example, while the UCH and MJD families do not have any pseudoenzyme, the JAMM family has the highest number with seven identified pseudo-DUBs. While lacking DUB activity, these proteins fulfil alternative roles such as allosterically activating other DUBs or serving as scaffold components within larger complexes. However, for some pseudo-DUBs, specific functions have not yet been identified (reviewed in detail by Walden et al., 2018).

Initially classified as pseudo-DUBs within the USP family, both USP39 and USP52 have been the subject of reconsideration (Walden et al., 2018). As previously discussed, several studies indicate that USP39 might exhibit DUB activity via non-conserved residues (Wu et al., 2019; Peng et al., 2020; Dong et al., 2021; Li et al., 2021; Chen et al., 2024), a hypothesis confirmed for USP52 (Yang et al., 2018). Both USP39 and USP52 lack the typical catalytic triad residues (cysteine, histidine and aspartate) found in other USPs. Interestingly, USP52 displays the ability to cleave various ubiquitin chain types when expressed in mammalian or insect cells, but not when produced in bacteria, suggesting that specific PTMs or protein folding might create a new active centre that enables ubiquitin hydrolysis (Yang et al., 2018). This discovery, combined with the emerging understanding that cysteine residues may contribute to the catalytic activity of certain DUBs but may be dispensable for it (Qiu et al., 2017), prompts a re-evaluation of whether initially labelled pseudo-DUBs truly lack catalytic function or whether the mechanisms by which they may hydrolyse ubiquitin or UBL proteins have not yet been discovered.

## 5 Concluding remarks

Ubiquitin and UBL modifications are involved in virtually all cellular functions and play very important roles in the proper functioning of the organism. Therefore, the regulation of these modifications at different levels in essential to prevent any disruptions. DUBs and UBL proteases exert their influence on these modifications primarily during precursor maturation and removal of ubiquitin or UBLs from substrates. Both, the overexpression and deficiency of these proteins have been associated with the development of numerous diseases. Contrary to catchy belief that attributes their significance solely to their catalytic function, it has been demonstrated that some DUBs and UBL proteases possess non-catalytic functions, which, in certain cases, are even more important for maintaining the homeostasis of the organism. A comprehensive understanding of the non-catalytic activities of these proteins could represent a major step towards the development of drugs and therapies for diseases involving these proteases as well as for predicting with greater precision the possible side effects they may cause. This review highlights currently known, non-catalytic activities of DUBs and UBL proteases and addresses their relevance.

The example of non-catalytic functions of USP18 highlights that even small proteases (USP18 is only 43 kDa), lacking additional characteristic domains, show pronounced moonlighting functions. This suggests that for DUBs, UBL proteases and enzymes in general, additional functions unrelated to the isopeptidase activity might be more frequent than expected even in non-multi domain proteins. It can be anticipated that more attentiveness for this phenomenon will unveil novel mechanisms for apparently well-characterized enzymes. In the case of USP18, its interaction with STAT2 even has diverse functions in different subcellular locations. While the STAT2-USP18 interaction secures negative regulation of type I IFN signalling close to the cell membrane, the same interaction affects target gene activation by restricting transcription via binding to regulatory DNA elements in the nucleus. Similar modes of action may introduce additional levels of regulation and complexity, and uncovering the underlying mechanisms and their physiological relevance will be intriguing. A compelling question for future research is to uncover the interplay between catalytic and non-catalytic functions. For instance, a DUB might serve essential scaffolding functions within a protein complex while simultaneously regulating ubiquitin/UBL deconjugation of proteins in close proximity. Binding to non-substrates might also influence the isopeptidase activity of DUBs. From an evolutionary standpoint, moonlighting functions dramatically expand the functional repertoire of the proteome, reminiscent of viral proteins that early on were described to encompass a range of functions in one polypeptide chain.

Moonlighting functions can be easily overlooked, especially in proteins clearly assigned to well-defined enzyme families, where functions may seem straightforward, and interpretations are often made without considering potential non-catalytic properties. Carefully planned experiments involving selective inactivation of only the catalytic core or rescue experiments with catalytically inactive variants are crucial to avoid misinterpretations.

Although pinpointing precise molecular mechanisms and structural-functional relationships may often be challenging, the discovery of more moonlighting functions emphasizes the need to approach the molecular mechanisms of DUBs and UBL proteases, in particular, and proteins, in general, without preconceptions. These new functions will enhance the overall understanding of various biological pathways and contribute to deciphering and addressing pathological abnormalities.

## References

[B1] Aichem A. Groettrup M. (2020). The ubiquitin-like modifier FAT10 – much more than a proteasome-targeting signal. J. Cell Sci. 133, jcs246041. 10.1242/jcs.246041 32719056

[B2] Akutsu M. Dikic I. Bremm A. (2016). Ubiquitin chain diversity at a glance. J. Cell Sci. 129, 875–880. 10.1242/jcs.183954 26906419

[B3] Amsterdam A. Nissen R. M. Sun Z. Swindell E. C. Farrington S. Hopkins N. (2004). Identification of 315 genes essential for early zebrafish development. Proc Natl Acad Sci U S A. 101, 12792-7. 10.1073/pnas.0403929101 15256591 PMC516474

[B4] Arimoto K. Miyauchi S. Troutman T. D. Zhang Y. Liu M. Stoner S. A. (2023). Expansion of interferon inducible gene pool via USP18 inhibition promotes cancer cell pyroptosis. Nat. Commun. 14, 251. 10.1038/s41467-022-35348-5 36646704 PMC9842760

[B5] Arimoto K. I. Löchte S. Stoner S. A. Burkart C. Zhang Y. Miyauchi S. (2017). STAT2 is an essential adaptor in USP18-mediated suppression of type i interferon signaling. Nat. Struct. Mol. Biol. 24, 279–289. 10.1038/nsmb.3378 28165510 PMC5365074

[B6] Aron R. Pellegrini P. Green E. W. Maddison D. C. Opoku-Nsiah K. Wong J. S. (2018). Deubiquitinase Usp12 functions noncatalytically to induce autophagy and confer neuroprotection in models of Huntington’s disease. Nat. Commun. 9, 3191. 10.1038/s41467-018-05653-z 30266909 PMC6162324

[B7] Baietti M. F. Simicek M. Abbasi Asbagh L. Radaelli E. Lievens S. Crowther J. (2016). OTUB 1 triggers lung cancer development by inhibiting RAS monoubiquitination. EMBO Mol. Med. 8, 288–303. 10.15252/emmm.201505972 26881969 PMC4772950

[B8] Bailey-Elkin B. A. Knaap R. C. M. Kikkert M. Mark B. L. (2017). Structure and function of viral deubiquitinating enzymes. J. Mol. Biol. 429, 3441–3470. 10.1016/j.jmb.2017.06.010 28625850 PMC7094624

[B9] Basar M. A. Beck D. B. Werner A. (2021). Deubiquitylases in developmental ubiquitin signaling and congenital diseases. Cell Death Differ. 28, 538–556. 10.1038/s41418-020-00697-5 33335288 PMC7862630

[B10] Basters A. Geurink P. P. Röcker A. Witting K. F. Tadayon R. Hess S. (2017). Structural basis of the specificity of USP18 toward ISG15. Nat. Struct. Mol. Biol. 24, 270–278. 10.1038/nsmb.3371 28165509 PMC5405867

[B11] Bosanac I. Wertz I. E. Pan B. Yu C. Kusam S. Lam C. (2010). Ubiquitin binding to A20 ZnF4 is required for modulation of NF-κB signaling. Mol. Cell 40, 548–557. 10.1016/j.molcel.2010.10.009 21095585

[B12] Brüninghoff K. Wulff S. Dörner W. Geiss-Friedlander R. Mootz H. D. (2022). A photo-crosslinking approach to identify class II SUMO-1 binders. Front. Chem. 10, 900989. 10.3389/fchem.2022.900989 35707458 PMC9191277

[B13] Burska U. L. Harle V. J. Coffey K. Darby S. Ramsey H. O’Neill D. (2013). Deubiquitinating enzyme Usp12 is a novel co-activator of the androgen receptor. J. Biol. Chem. 288, 32641–32650. 10.1074/jbc.M113.485912 24056413 PMC3820899

[B14] Castagnoli L. Mandaliti W. Nepravishta R. Valentini E. Mattioni A. Procopio R. (2019). Selectivity of the CUBAN domain in the recognition of ubiquitin and NEDD8. FEBS J. 286, 653–677. 10.1111/febs.14752 30659753

[B15] Chang H. M. Yeh E. T. H. (2020a). Sumo: from bench to bedside. Physiol. Rev. 100, 1599–1619. 10.1152/physrev.00025.2019 32666886 PMC7717128

[B16] Chang H.-M. Yeh E. T. H. (2020b). SUMO: from bench to bedside. Physiol. Rev. 100, 1599–1619. 10.1152/physrev.00025.2019 32666886 PMC7717128

[B17] Chen P. C. Bhattacharyya B. J. Hanna J. Minkel H. Wilson J. A. Finley D. (2011). Ubiquitin homeostasis is critical for synaptic development and function. J. Neurosci. 31, 17505–17513. 10.1523/JNEUROSCI.2922-11.2011 22131412 PMC3253363

[B18] Chen P. C. Qin L. N. Li X. M. Walters B. J. Wilson J. A. Mei L. (2009). The proteasome-associated deubiquitinating enzyme Usp14 is essential for the maintenance of synaptic ubiquitin levels and the development of neuromuscular junctions. J. Neurosci. 29, 10909–10919. 10.1523/JNEUROSCI.2635-09.2009 19726649 PMC2766780

[B19] Chen X. Tian L. Zhang L. Gao W. Yu M. Li Z. (2024). Deubiquitinase USP39 promotes SARS-CoV-2 replication by deubiquitinating and stabilizing the envelope protein. Antivir. Res. 221, 105790. 10.1016/j.antiviral.2023.105790 38158131

[B20] Chen Y. Li Y. Dai R. S. Savage J. C. Shinde U. Klimek J. (2023). The ubiquitin-specific protease USP36 SUMOylates EXOSC10 and promotes the nucleolar RNA exosome function in rRNA processing. Nucleic Acids Res. 51, 3934–3949. 10.1093/nar/gkad140 36912080 PMC10164564

[B21] Clague M. J. Urbé S. Komander D. (2019). Breaking the chains: deubiquitylating enzyme specificity begets function. Nat. Rev. Mol. Cell Biol. 20, 338–352. 10.1038/s41580-019-0099-1 30733604

[B22] Cui D. Wang Z. Dang Q. Wang J. Qin J. Song J. (2023). Spliceosome component Usp39 contributes to hepatic lipid homeostasis through the regulation of autophagy. Nat. Commun. 14, 7032. 10.1038/s41467-023-42461-6 37923718 PMC10624899

[B23] Damgaard R. B. (2021). The ubiquitin system: from cell signalling to disease biology and new therapeutic opportunities. Cell Death Differ. 28, 423–426. 10.1038/s41418-020-00703-w 33446876 PMC7862391

[B24] Das R. Schwintzer L. Vinopal S. Roca E. A. Sylvester M. Oprisoreanu A. M. (2019). New roles for the de-ubiquitylating enzyme OTUD4 in an RNA-protein network and RNA granules. J. Cell Sci. 132, jcs229252. 10.1242/jcs.229252 31138677 PMC6602300

[B25] De A. Dainichi T. Rathinam C. V. Ghosh S. (2014). The deubiquitinase activity of A 20 is dispensable for NF ‐κ B signaling. EMBO Rep. 15, 775–783. 10.15252/embr.201338305 24878851 PMC4196981

[B26] Deng H. Jia S. Tang J. Rong F. Xu C. Chen X. (2023). SET7 methylates the deubiquitinase OTUB1 at Lys 122 to impair its binding to E2 enzyme UBC13 and relieve its suppressive role on ferroptosis. J. Biol. Chem. 299, 103054. 10.1016/j.jbc.2023.103054 36822329 PMC10040876

[B27] Dikic I. Schulman B. A. (2022). An expanded lexicon for the ubiquitin code. Nat. Rev. Mol. Cell Biol. 24, 273–287. 10.1038/s41580-022-00543-1 36284179 PMC9595094

[B28] Dong X. Liu Z. Zhang E. Zhang P. Wang Y. Hang J. (2021). USP39 promotes tumorigenesis by stabilizing and deubiquitinating SP1 protein in hepatocellular carcinoma. Cell Signal 85, 110068. 10.1016/j.cellsig.2021.110068 34197957

[B29] Duncan C. J. A. Thompson B. J. Chen R. Rice G. I. Gothe F. Young D. F. (2019). Severe type I interferonopathy and unrestrained interferon signaling due to a homozygous germline mutation in STAT2. Sci. Immunol. 4, eaav7501. 10.1126/sciimmunol.aav7501 31836668 PMC7115903

[B30] Elliott P. R. Leske D. Wagstaff J. Schlicher L. Berridge G. Maslen S. (2021). Regulation of CYLD activity and specificity by phosphorylation and ubiquitin-binding CAP-Gly domains. Cell Rep. 37, 109777. 10.1016/j.celrep.2021.109777 34610306 PMC8511506

[B31] Erven I. Abraham E. Hermanns T. Baumann U. Hofmann K. (2022). A widely distributed family of eukaryotic and bacterial deubiquitinases related to herpesviral large tegument proteins. Nat. Commun. 13, 7643. 10.1038/s41467-022-35244-y 36496440 PMC9741609

[B32] Esposito M. Akman H. B. Giron P. Ceregido M. A. Schepers R. Ramos Paez L. C. (2020). USP13 controls the stability of Aurora B impacting progression through the cell cycle. Oncogene 39, 6009–6023. 10.1038/s41388-020-01396-8 32772043

[B33] Fan G. Wang F. Chen Y. Zheng Q. Xiong J. Lv Q. (2023). The deubiquitinase OTUD1 noncanonically suppresses Akt activation through its N-terminal intrinsically disordered region. Cell Rep. 42, 111916. 10.1016/j.celrep.2022.111916 36640312

[B34] Fan J. B. Arimoto K. L. Motamedchaboki K. Yan M. Wolf D. A. Zhang D. E. (2015). Identification and characterization of a novel ISG15-ubiquitin mixed chain and its role in regulating protein homeostasis. Sci. Rep. 5, 12704. 10.1038/srep12704 26226047 PMC4520236

[B35] Flierman D. Van Der Heden Van Noort G. J. Ekkebus R. Geurink P. P. Mevissen T. E. T. Hospenthal M. K. (2016). Non-hydrolyzable diubiquitin probes reveal linkage-specific reactivity of deubiquitylating enzymes mediated by S2 pockets. Cell Chem. Biol. 23, 472–482. 10.1016/j.chembiol.2016.03.009 27066941 PMC4850247

[B36] French M. E. Koehler C. F. Hunter T. (2021). Emerging functions of branched ubiquitin chains. Cell Discov. 7, 6. 10.1038/s41421-020-00237-y 33495455 PMC7835216

[B37] Gangula N. R. Maddika S. (2013). WD repeat protein WDR48 in complex with deubiquitinase USP12 suppresses akt-dependent cell survival signaling by stabilizing ph domain leucine-rich repeat protein phosphatase 1 (PHLPP1). J. Biol. Chem. 288, 34545–34554. 10.1074/jbc.M113.503383 24145035 PMC3843068

[B38] Gärtner A. Wagner K. Hölper S. Kunz K. Rodriguez M. S. Müller S. (2018). Acetylation of SUMO 2 at lysine 11 favors the formation of non‐canonical SUMO chains. EMBO Rep. 19, e46117. 10.15252/embr.201846117 30201799 PMC6216285

[B39] Garvin A. J. Densham R. M. Blair-Reid S. A. Pratt K. M. Stone H. R. Weekes D. (2013). The deSUMOylase SENP7 promotes chromatin relaxation for homologous recombination DNA repair. EMBO Rep. 14, 975–983. 10.1038/embor.2013.141 24018422 PMC3818072

[B40] González-Prieto R. Eifler-Olivi K. Claessens L. A. Willemstein E. Xiao Z. Talavera Ormeno C. M. P. (2021). Global non-covalent SUMO interaction networks reveal SUMO-dependent stabilization of the non-homologous end joining complex. Cell Rep. 34, 108691. 10.1016/j.celrep.2021.108691 33503430

[B41] Grou C. P. Pinto M. P. Mendes A. V. Domingues P. Azevedo J. E. (2015). The *de novo* synthesis of ubiquitin: identification of deubiquitinases acting on ubiquitin precursors. Sci. Rep. 5, 12836. 10.1038/srep12836 26235645 PMC4522658

[B42] Gruber C. Martin-Fernandez M. Ailal F. Qiu X. Taft J. Altman J. (2020). Homozygous STAT2 gain-of-function mutation by loss of USP18 activity in a patient with type I interferonopathy. J. Exp. Med. 217, e20192319. 10.1084/jem.20192319 32092142 PMC7201920

[B43] Hadjivassiliou H. Rosenberg O. S. Guthrie C. (2014). The crystal structure of *S. cerevisiae* Sad1, a catalytically inactive deubiquitinase that is broadly required for pre-mRNA splicing. RNA 20, 656–669. 10.1261/rna.042838.113 24681967 PMC3988567

[B44] Han C. Yang L. Choi H. H. Baddour J. Achreja A. Liu Y. (2016). Amplification of USP13 drives ovarian cancer metabolism. Nat. Commun. 7, 13525. 10.1038/ncomms13525 27892457 PMC5133706

[B45] Hanna J. Hathaway N. A. Tone Y. Crosas B. Elsasser S. Kirkpatrick D. S. S. (2006). Deubiquitinating enzyme Ubp6 functions noncatalytically to delay proteasomal degradation. Cell 127, 99–111. 10.1016/j.cell.2006.07.038 17018280

[B46] Harakandi C. Nininahazwe L. Xu H. Liu B. He C. Zheng Y. C. (2021). Recent advances on the intervention sites targeting USP7-MDM2-p53 in cancer therapy. Bioorg Chem. 116, 105273. 10.1016/j.bioorg.2021.105273 34474304

[B47] Hayden M. S. Ghosh S. (2004). Signaling to NF-kappaB. Genes Dev. 18, 2195–2224. 10.1101/gad.1228704 15371334

[B48] Heger K. Wickliffe K. E. Ndoja A. Zhang J. Murthy A. Dugger D. L. (2018). OTULIN limits cell death and inflammation by deubiquitinating LUBAC. Nature 559, 120–124. 10.1038/s41586-018-0256-2 29950720

[B49] Herhaus L. Al-Salihi M. MacArtney T. Weidlich S. Sapkota G. P. (2013). OTUB1 enhances TGFβ signalling by inhibiting the ubiquitylation and degradation of active SMAD2/3. Nat. Commun. 4, 2519. 10.1038/ncomms3519 24071738 PMC3791481

[B50] Hermanns T. Pichlo C. Woiwode I. Klopffleisch K. Witting K. F. Ovaa H. (2018). A family of unconventional deubiquitinases with modular chain specificity determinants. Nat. Commun. 9, 799. 10.1038/s41467-018-03148-5 29476094 PMC5824887

[B51] Hochstrasser M. (2009). Origin and function of ubiquitin-like proteins. Nature 458, 422–429. 10.1038/nature07958 19325621 PMC2819001

[B52] Honke N. Shaabani N. Zhang D. E. Hardt C. Lang K. S. (2016). Multiple functions of USP18. Cell Death Dis. 7, e2444. 10.1038/cddis.2016.326 27809302 PMC5260889

[B53] Huang Y.-H. Chung C.-S. Kao D.-I. Kao T.-C. Cheng S.-C. (2014). Sad1 counteracts brr2-mediated dissociation of U4/U6.U5 in tri-snRNP homeostasis. Mol. Cell Biol. 34, 210–220. 10.1128/mcb.00837-13 24190974 PMC3911299

[B54] Hung K. Y. S. Klumpe S. Eisele M. R. Elsasser S. Tian G. Sun S. (2022). Allosteric control of Ubp6 and the proteasome via a bidirectional switch. Nat. Commun. 13, 838. 10.1038/s41467-022-28186-y 35149681 PMC8837689

[B55] Hutten S. Chachami G. Winter U. Melchior F. Lamond A. I. (2014). A role for the Cajal-body-associated SUMO isopeptidase USPL1 in snRNA transcription mediated by RNA polymerase II. J. Cell Sci. 127, 1065–1078. 10.1242/jcs.141788 24413172 PMC3937775

[B56] Hwang J. T. Lee A. Kho C. (2022). Ubiquitin and ubiquitin-like proteins in cancer, neurodegenerative disorders, and heart diseases. Int. J. Mol. Sci. 23, 5053. 10.3390/ijms23095053 35563444 PMC9105348

[B57] Isaacson M. K. Ploegh H. L. (2009). Ubiquitination, ubiquitin-like modifiers, and deubiquitination in viral infection. Cell Host Microbe 5, 559–570. 10.1016/j.chom.2009.05.012 19527883 PMC7103382

[B58] Janisiw E. Raices M. Balmir F. Paulin L. F. Baudrimont A. von Haeseler A. (2020). Poly(ADP-ribose) glycohydrolase coordinates meiotic DNA double-strand break induction and repair independent of its catalytic activity. Nat. Commun. 11, 4869. 10.1038/s41467-020-18693-1 32978394 PMC7519143

[B59] Jansen N. S. Vertegaal A. C. O. (2021). A chain of events: regulating target proteins by SUMO polymers. Trends Biochem. Sci. 46, 113–123. 10.1016/j.tibs.2020.09.002 33008689

[B60] Jaynes P. W. Iyengar P. V. Lui S. K. L. Tan T. Z. Vasilevski N. Wright S. C. E. (2020). OTUD4 enhances TGFβ signalling through regulation of the TGFβ receptor complex. Sci. Rep. 10, 15725. 10.1038/s41598-020-72791-0 32973272 PMC7519109

[B61] Joo H. Y. Jones A. Yang C. Zhai L. Smith IV A. D. Zhang Z. (2011). Regulation of histone H2A and H2B deubiquitination and xenopus development by USP12 and USP46. J. Biol. Chem. 286, 7190–7201. 10.1074/jbc.M110.158311 21183687 PMC3044976

[B62] Juang Y. C. Landry M. C. Sanches M. Vittal V. Leung C. C. Y. Ceccarelli D. F. (2012). OTUB1 Co-opts lys48-linked ubiquitin recognition to suppress E2 enzyme function. Mol. Cell 45, 384–397. 10.1016/j.molcel.2012.01.011 22325355 PMC3306812

[B63] Juncker M. Kim C. Reed R. Haas A. Schwartzenburg J. Desai S. (2021). ISG15 attenuates post-translational modifications of mitofusins and congression of damaged mitochondria in Ataxia Telangiectasia cells. Biochimica Biophysica Acta - Mol. Basis Dis. 1867, 166102. 10.1016/j.bbadis.2021.166102 33617986

[B64] Kedia S. Aghanoori M. R. Burns K. M. L. Subha M. Williams L. Wen P. (2022). Ubiquitination and deubiquitination of 4E-T regulate neural progenitor cell maintenance and neurogenesis by controlling P-body formation. Cell Rep. 40, 111070. 10.1016/j.celrep.2022.111070 35830814

[B65] Keiten-Schmitz J. Schunck K. Müller S. (2020). SUMO chains rule on chromatin occupancy. Front. Cell Dev. Biol. 7, 343. 10.3389/fcell.2019.00343 31998715 PMC6965010

[B66] Kelsall I. R. (2022). Non-lysine ubiquitylation: doing things differently. Front. Mol. Biosci. 9, 1008175. 10.3389/fmolb.2022.1008175 36200073 PMC9527308

[B67] Kerscher O. Felberbaum R. Hochstrasser M. (2006). Modification of proteins by ubiquitin and ubiquitin-like proteins. Annu. Rev. Cell Dev. Biol. 22, 159–180. 10.1146/annurev.cellbio.22.010605.093503 16753028

[B68] Ketscher L. Hannß R. Moralese D. J. Basters A. Guerra S. Goldmann T. (2015). Selective inactivation of USP18 isopeptidase activity *in vivo* enhances ISG15 conjugation and viral resistance. Proc. Natl. Acad. Sci. U. S. A. 112, 1577–1582. 10.1073/pnas.1412881112 25605921 PMC4321242

[B69] Kim H. T. Goldberg A. L. (2017). The deubiquitinating enzyme Usp14 allosterically inhibits multiple proteasomal activities and ubiquitin-independent proteolysis. J. Biol. Chem. 292, 9830–9839. 10.1074/jbc.M116.763128 28416611 PMC5465503

[B70] Kim J. J. Lee S. Y. Hwang Y. Kim S. Chung J. M. Park S. (2021a). USP39 promotes non-homologous end-joining repair by poly(ADP-ribose)-induced liquid demixing. Nucleic Acids Res. 49, 11083–11102. 10.1093/nar/gkab892 34614178 PMC8565343

[B71] Kim K.Il Malakhova O. A. Hoebe K. Yan M. Beutler B. Zhang D.-E. (2005). Enhanced antibacterial potential in UBP43-deficient mice against *Salmonella typhimurium* infection by up-regulating type I IFN signaling. J. Immunol. 175, 847–854. 10.4049/jimmunol.175.2.847 16002682

[B72] Kim W. Zhao F. Gao H. Qin S. Hou J. Deng M. (2021b). USP13 regulates the replication stress response by deubiquitinating TopBP1. DNA Repair (Amst) 100, 103063. 10.1016/j.dnarep.2021.103063 33592542 PMC7951086

[B73] Kimura-Yoshida C. Mochida K. Kanno S. I. Matsuo I. (2022). USP39 is essential for mammalian epithelial morphogenesis through upregulation of planar cell polarity components. Commun. Biol. 5, 378. 10.1038/s42003-022-03254-7 35440748 PMC9018712

[B74] Kitao T. Nagai H. Kubori T. (2020). Divergence of Legionella effectors reversing conventional and unconventional ubiquitination. Front. Cell Infect. Microbiol. 10, 448. 10.3389/fcimb.2020.00448 32974222 PMC7472693

[B75] Klemm T. Ebert G. Calleja D. J. Allison C. C. Richardson L. W. Bernardini J. P. (2020). Mechanism and inhibition of the papain‐like protease, PLpro, of SARS‐CoV‐2. EMBO J. 39, e106275. 10.15252/embj.2020106275 32845033 PMC7461020

[B76] Knobeloch K.-P. Utermöhlen O. Kisser A. Prinz M. Horak I. (2005). Reexamination of the role of ubiquitin-like modifier ISG15 in the phenotype of UBP43-deficient mice. Mol. Cell Biol. 25, 11030–11034. 10.1128/mcb.25.24.11030-11034.2005 16314524 PMC1316970

[B77] Kolla S. D. D. Ye M. Mark K. G. Rapé M. (2022). Assembly and function of branched ubiquitin chains. Trends Biochem. Sci. 47, 759–771. 10.1016/j.tibs.2022.04.003 35508449

[B78] Komander D. Rape M. (2012). The ubiquitin code. Annu. Rev. Biochem. 81, 203–229. 10.1146/annurev-biochem-060310-170328 22524316

[B79] Kon N. Kobayashi Y. Li M. Brooks C. L. Ludwig T. Gu W. (2010). Inactivation of HAUSP *in vivo* modulates p53 function. Oncogene 29, 1270–1279. 10.1038/onc.2009.427 19946331 PMC2857765

[B80] Krikos A. Laherty C. D. Dixit V. M. (1992). Transcriptional activation of the tumor necrosis factor alpha-inducible zinc finger protein, A20, is mediated by kappa B elements. J. Biol. Chem. 267, 17971–17976. 10.1016/s0021-9258(19)37138-8 1381359

[B81] Kunz K. Piller T. Müller S. (2018). SUMO-specific proteases and isopeptidases of the SENP family at a glance. J. Cell Sci. 131, jcs211904. 10.1242/jcs.211904 29559551

[B82] Lange S. M. Armstrong L. A. Kulathu Y. (2022). Deubiquitinases: from mechanisms to their inhibition by small molecules. Mol. Cell 82, 15–29. 10.1016/j.molcel.2021.10.027 34813758

[B83] Lee E. G. Boone D. L. Chai S. Libby S. L. Chien M. Lodolce J. P. (2000). Failure to regulate TNF-induced NF-kappaB and cell death responses in A20-deficient mice. Science. 289, 2350–2354. 10.1126/science.289.5488.2350 11009421 PMC3582399

[B84] Li X. Yang G. Zhang W. Qin B. Ye Z. Shi H. (2022a). USP13: multiple functions and target inhibition. Front. Cell Dev. Biol. 10, 875124. 10.3389/fcell.2022.875124 35445009 PMC9014248

[B85] Li X. Yuan J. Song C. Lei Y. Xu J. Zhang G. (2021). Deubiquitinase USP39 and E3 ligase TRIM26 balance the level of ZEB1 ubiquitination and thereby determine the progression of hepatocellular carcinoma. Cell Death Differ. 28, 2315–2332. 10.1038/s41418-021-00754-7 33649471 PMC8329202

[B86] Li Y. Luo K. Yin Y. Wu C. Deng M. Li L. (2017). USP13 regulates the RAP80-BRCA1 complex dependent DNA damage response. Nat. Commun. 8, 15752. 10.1038/ncomms15752 28569838 PMC5461494

[B87] Li Y. Varejão N. Reverter D. (2022b). Structural basis for the SUMO protease activity of the atypical ubiquitin-specific protease USPL1. Nat. Commun. 13, 1819. 10.1038/s41467-022-29485-0 35383180 PMC8983731

[B88] Liu C. Sun L. Tan Y. Wang Q. Luo T. Li C. (2023). USP7 represses lineage differentiation genes in mouse embryonic stem cells by both catalytic and noncatalytic activities. Sci Adv. 9, 20. 10.1126/sciadv.ade3888 PMC10191441 37196079

[B89] Liu J. Jin L. Chen X. Yuan Y. Zuo Y. Miao Y. (2020). USP12 translocation maintains interferon antiviral efficacy by inhibiting CBP acetyltransferase activity. PLoS Pathog. 16, e1008215. 10.1371/journal.ppat.1008215 31899788 PMC6961928

[B90] Liu X. Deng H. Tang J. Wang Z. Zhu C. Cai X. (2022). OTUB1 augments hypoxia signaling via its non-canonical ubiquitination inhibition of HIF-1α during hypoxia adaptation. Cell Death Dis. 13, 560. 10.1038/s41419-022-05008-z 35732631 PMC9217984

[B91] Liu Y. Soetandyo N. Lee J. Liu L. Xu Y. Clemons W. M. (2014). USP13 antagonizes gp78 to maintain functionality of a chaperone in ER-associated degradation. Elife 3, e01369. 10.7554/elife.01369 24424410 PMC3889402

[B92] Lobato-Gil S. Heidelberger J. B. Maghames C. Bailly A. Brunello L. Rodriguez M. S. (2021). Proteome-wide identification of NEDD8 modification sites reveals distinct proteomes for canonical and atypical NEDDylation. Cell Rep. 34, 108635. 10.1016/j.celrep.2020.108635 33472076

[B93] Lork M. Verhelst K. Beyaert R. (2017). CYLD, A20 and OTULIN deubiquitinases in NF-κB signaling and cell death: so similar, yet so different. Cell Death Differ. 24, 1172–1183. 10.1038/cdd.2017.46 28362430 PMC5520167

[B94] Lu T. T. Onizawa M. Hammer G. E. Turer E. E. Yin Q. Damko E. (2013). Dimerization and ubiquitin mediated recruitment of A20, a complex deubiquitinating enzyme. Immunity 38, 896–905. 10.1016/j.immuni.2013.03.008 23602765 PMC3665706

[B95] Makarova O. V. Makarov E. M. Lührmann R. (2001). The 65 and 110 kDa SR-related proteins of the U4/U6U5 tri-snRNP are essential for the assembly of mature spliceosomes. EMBO J. 20, 2553–2563. 10.1093/emboj/20.10.2553 11350945 PMC125249

[B96] Malakhova O. A. Kim K.Il Luo J. K. Zou W. Kumar K. G. S. Fuchs S. Y. (2006). UBP43 is a novel regulator of interferon signaling independent of its ISG15 isopeptidase activity. EMBO J. 25, 2358–2367. 10.1038/sj.emboj.7601149 16710296 PMC1478183

[B97] Malakhova O. A. Yan M. Malakhov M. P. Yuan Y. Ritchie K. J. Kim K.Il (2003). Protein ISGylation modulates the JAK-STAT signaling pathway. Genes Dev. 17, 455–460. 10.1101/gad.1056303 12600939 PMC195994

[B98] Martín-Fernandez M. Buta S. Le Voyer T. Li Z. Dynesen L. T. Vuillier F. (2022). A partial form of inherited human USP18 deficiency underlies infection and inflammation. J. Exp. Med. 219, e20211273. 10.1084/jem.20211273 35258551 PMC8908790

[B99] Martín-Villanueva S. Gutiérrez G. Kressler D. de la Cruz J. (2021). Ubiquitin and ubiquitin-like proteins and domains in ribosome production and function: chance or necessity? Int. J. Mol. Sci. 22, 4359. 10.3390/ijms22094359 33921964 PMC8122580

[B100] Matic I. Schimmel J. Hendriks I. A. van Santen M. A. van de Rijke F. van Dam H. (2010). Site-specific identification of SUMO-2 targets in cells reveals an inverted SUMOylation motif and a hydrophobic cluster SUMOylation motif. Mol. Cell 39, 641–652. 10.1016/j.molcel.2010.07.026 20797634

[B101] Matic I. van Hagen M. Schimmel J. Macek B. Ogg S. C. Tatham M. H. (2008). *In vivo* identification of human small ubiquitin-like modifier polymerization sites by high accuracy mass spectrometry and an *in vitro* to *in vivo* strategy. Mol. Cell. Proteomics 7, 132–144. 10.1074/mcp.M700173-MCP200 17938407 PMC3840926

[B102] McClurg U. L. Chit N. C. T. H. Azizyan M. Edwards J. Nabbi A. Riabowol K. T. (2018). Molecular mechanism of the TP53-MDM2-AR-AKT signalling network regulation by USP12. Oncogene 37, 4679–4691. 10.1038/s41388-018-0283-3 29755129

[B103] Meszka I. Polanowska J. Xirodimas D. P. (2022). Mixed in chains: NEDD8 polymers in the protein quality control system. Semin. Cell Dev. Biol. 132, 27–37. 10.1016/j.semcdb.2022.01.005 35078718

[B104] Meuwissen M. E. C. Schot R. Buta S. Oudesluijs G. Tinschert S. Speer S. D. (2016). Human USP18 deficiency underlies type 1 interferonopathy leading to severe pseudo-TOR CH syndrome. J. Exp. Med. 213, 1163–1174. 10.1084/jem.20151529 27325888 PMC4925017

[B105] Mevissen T. E. T. Komander D. (2017). Mechanisms of deubiquitinase specificity and regulation. Annu Rev Biochem. 86, 159-192. 10.1146/annurev-biochem 28498721

[B106] Millrine D. Peter J. J. Kulathu Y. (2023). A guide to UFMylation, an emerging posttranslational modification. FEBS J. 290, 5040–5056. 10.1111/febs.16730 36680403 PMC10952357

[B107] Miyauchi S. Arimotoichiro K. Liu M. Zhang Y. Zhang D. E. (2023). Reprogramming of tumor-associated macrophages via NEDD4-mediated CSF1R degradation by targeting USP18. Cell Rep. 42, 113560. 10.1016/j.celrep.2023.113560 38100351 PMC10822669

[B108] Moretti J. Chastagner P. Liang C. C. Cohn M. A. Israël A. Brou C. (2012). The ubiquitin-specific protease 12 (USP12) is a negative regulator of notch signaling acting on notch receptor trafficking toward degradation. J. Biol. Chem. 287, 29429–29441. 10.1074/jbc.M112.366807 22778262 PMC3436160

[B109] Morrell R. Sadanandom A. (2019). Dealing with stress: a review of plant SUMO proteases. Front. Plant Sci. 10, 1122. 10.3389/fpls.2019.01122 31620153 PMC6759571

[B110] Morrow M. E. Morgan M. T. Clerici M. Growkova K. Yan M. Komander D. (2018). Active site alanine mutations convert deubiquitinases into high‐affinity ubiquitin‐binding proteins. EMBO Rep. 19, e45680. 10.15252/embr.201745680 30150323 PMC6172466

[B111] Mustachio L. M. Lu Y. Kawakami M. Roszik J. Freemantle S. J. Liu X. (2018). Evidence for the ISG15-specific deubiquitinase usp18 as an antineoplastic target. Cancer Res. 78, 587–592. 10.1158/0008-5472.CAN-17-1752 29343520 PMC6080720

[B112] Nakada S. Tai I. Panier S. Al-Hakim A. Iemura S. I. Juang Y. C. (2010). Non-canonical inhibition of DNA damage-dependent ubiquitination by OTUB1. Nature 466, 941–946. 10.1038/nature09297 20725033

[B113] Napolitano A. van der Veen A. G. Bunyan M. Borg A. Frith D. Howell S. (2018). Cysteine-reactive free ISG15 generates IL-1β-producing CD8α+ dendritic cells at the site of infection. J. Immunol. 201, 604–614. 10.4049/jimmunol.1701322 29891555 PMC6036233

[B114] Nayak A. Müller S. (2014). SUMO-specific proteases/isopeptidases: SENPs and beyond. Genome Biol. 15, 422. 10.1186/s13059-014-0422-2 25315341 PMC4281951

[B115] Niu K. Shi Y. Lv Q. Wang Y. Chen J. Zhang W. (2023). Spotlights on ubiquitin-specific protease 12 (USP12) in diseases: from multifaceted roles to pathophysiological mechanisms. J. Transl. Med. 21, 665. 10.1186/s12967-023-04540-6 37752518 PMC10521459

[B116] O’Dea R. Kazi N. Hoffmann-Benito A. Zhao Z. Recknagel S. Wendrich K. (2023). Molecular basis for ubiquitin/Fubi cross-reactivity in USP16 and USP36. Nat. Chem. Biol. 19, 1394–1405. 10.1038/s41589-023-01388-1 37443395 PMC10611586

[B117] Ohki Y. Funatsu N. Konishi N. Chiba T. (2009). The mechanism of poly-NEDD8 chain formation *in vitro* . Biochem. Biophys. Res. Commun. 381, 443–447. 10.1016/j.bbrc.2009.02.090 19245792

[B118] Okumura F. Lenschow D. J. Zhang D. E. (2008). Nitrosylation of ISG15 prevents the disulfide bond-mediated dimerization of ISG15 and contributes to effective ISGylation. J. Biol. Chem. 283, 24484–24488. 10.1074/jbc.M803795200 18606809 PMC2528991

[B119] Ozhelvaci F. Steczkiewicz K. (2023). Identification and classification of papain-like cysteine proteinases. J. Biol. Chem. 299, 104801. 10.1016/j.jbc.2023.104801 37164157 PMC10318531

[B120] Pang Y. Yamamoto H. Sakamoto H. Oku M. Mutungi J. K. Sahani M. H. (2019). Evolution from covalent conjugation to non-covalent interaction in the ubiquitin-like ATG12 system. Nat. Struct. Mol. Biol. 26, 289–296. 10.1038/s41594-019-0204-3 30911187

[B121] Pasupala N. Morrow M. E. Que L. T. Malynn B. A. Ma A. Wolberger C. (2018). OTUB1 non-catalytically stabilizes the E2 ubiquitin-conjugating enzyme UBE2E1 by preventing its autoubiquitination. J. Biol. Chem. 293, 18285–18295. 10.1074/jbc.RA118.004677 30282802 PMC6254341

[B122] Peng Y. Guo J. Sun T. Fu Y. Zheng H. Dong C. (2020). USP39 serves as a deubiquitinase to stabilize STAT1 and sustains type I IFN–induced antiviral immunity. J. Immunol. 205, 3167–3178. 10.4049/jimmunol.1901384 33127822

[B123] Pérez Berrocal D. A. Witting K. F. Ovaa H. Mulder M. P. C. (2020). Hybrid chains: a collaboration of ubiquitin and ubiquitin-like modifiers introducing cross-functionality to the ubiquitin code. Front. Chem. 7, 931. 10.3389/fchem.2019.00931 32039151 PMC6987259

[B124] Perng Y. C. Lenschow D. J. (2018). ISG15 in antiviral immunity and beyond. Nat. Rev. Microbiol. 16, 423–439. 10.1038/s41579-018-0020-5 29769653 PMC7097117

[B125] Peter J. J. Magnussen H. M. DaRosa P. A. Millrine D. Matthews S. P. Lamoliatte F. (2022). A non‐canonical scaffold‐type E3 ligase complex mediates protein UFMylation. EMBO J. 41, e111015. 10.15252/embj.2022111015 36121123 PMC9627666

[B126] Polykratis A. Martens A. Eren R. O. Shirasaki Y. Yamagishi M. Yamaguchi Y. (2019). A20 prevents inflammasome-dependent arthritis by inhibiting macrophage necroptosis through its ZnF7 ubiquitin-binding domain. Nat. Cell Biol. 21, 731–742. 10.1038/s41556-019-0324-3 31086261

[B127] Pruneda J. N. Durkin C. H. Geurink P. P. Ovaa H. Santhanam B. Holden D. W. (2016). The molecular basis for ubiquitin and ubiquitin-like specificities in bacterial effector proteases. Mol. Cell 63, 261–276. 10.1016/j.molcel.2016.06.015 27425412 PMC4961225

[B128] Qiu J. Yu K. Fei X. Liu Y. Nakayasu E. S. Piehowski P. D. (2017). A unique deubiquitinase that deconjugates phosphoribosyl-linked protein ubiquitination. Cell Res. 27, 865–881. 10.1038/cr.2017.66 28497808 PMC5518988

[B129] Randow F. Lehner P. (2009). Viral avoidance and exploitation of the ubiquitin system. Nat. Cell Biol. 11, 527–534. 10.1038/ncb0509-527 19404332

[B130] Rauch J. Volinsky N. Romano D. Kolch W. (2011). The secret life of kinases: functions beyond catalysis. Cell Commun. Signal. 9, 23. 10.1186/1478-811X-9-23 22035226 PMC3215182

[B131] Ravichandran K. E. Kaduhr L. Skupien‐Rabian B. Shvetsova E. Sokołowski M. Krutyhołowa R. (2022). E2/E3 ‐independent ubiquitin‐like protein conjugation by Urm1 is directly coupled to cysteine persulfidation. EMBO J. 41, e111318. 10.15252/embj.2022111318 36102610 PMC9574740

[B132] Rawlings N. D. Barrett A. J. (2013). Introduction: the clans and families of cysteine peptidases. Handb. Proteolytic Enzym., 1743–1773. 10.1016/B978-0-12-382219-2.00404-X

[B133] Rawlings N. D. Barrett A. J. Thomas P. D. Huang X. Bateman A. Finn R. D. (2018). The MEROPS database of proteolytic enzymes, their substrates and inhibitors in 2017 and a comparison with peptidases in the PANTHER database. Nucleic Acids Res. 46, D624–D632. 10.1093/nar/gkx1134 29145643 PMC5753285

[B134] Razani B. Whang M. I. Kim F. S. Nakamura M. C. Sun X. Advincula R. (2020). Non-catalytic ubiquitin binding by A20 prevents psoriatic arthritis–like disease and inflammation. Nat. Immunol. 21, 422–433. 10.1038/s41590-020-0634-4 32205880 PMC7195210

[B135] Reverter D. Wu K. Erdene T. G. Pan Z. Q. Wilkinson K. D. Lima C. D. (2005). Structure of a complex between Nedd8 and the Ulp/Senp protease family member Den1. J. Mol. Biol. 345, 141–151. 10.1016/j.jmb.2004.10.022 15567417

[B136] Ritchie K. J. Malakhov M. P. Hetherington C. J. Zhou L. Little M. T. Malakhova O. A. (2002). Dysregulation of protein modification by ISG15 results in brain cell injury. Genes Dev. 16, 2207–2212. 10.1101/gad.1010202 12208842 PMC186669

[B137] Roberts C. G. Franklin T. G. Pruneda J. N. (2023). Ubiquitin‐targeted bacterial effectors: rule breakers of the ubiquitin system. EMBO J. 42, e114318. 10.15252/embj.2023114318 37555693 PMC10505922

[B138] Ronau J. A. Beckmann J. F. Hochstrasser M. (2016). Substrate specificity of the ubiquitin and Ubl proteases. Cell Res. 26, 441–456. 10.1038/cr.2016.38 27012468 PMC4822132

[B139] Rougé L. Bainbridge T. W. Kwok M. Tong R. Di Lello P. Wertz I. E. (2016). Molecular understanding of USP7 substrate recognition and C-terminal activation. Structure 24, 1335–1345. 10.1016/j.str.2016.05.020 27452404

[B140] Ruan G. X. Li Y. Chen W. Huang H. Zhang R. Chen C. (2022). The spliceosome component Usp39 controls B cell development by regulating immunoglobulin gene rearrangement. Cell Rep. 38, 110338. 10.1016/j.celrep.2022.110338 35139388

[B141] Ruiz‐serrano A. Boyle C. N. Rodríguez J. M. M. Günter J. Jucht A. E. Pfundstein S. (2022). The deubiquitinase OTUB1 is a key regulator of energy metabolism. Int. J. Mol. Sci. 23, 1536. 10.3390/ijms23031536 35163456 PMC8836018

[B142] Ruiz-Serrano A. Monné Rodríguez J. M. Günter J. Sherman S. P. M. Jucht A. E. Fluechter P. (2021). OTUB1 regulates lung development, adult lung tissue homeostasis, and respiratory control. FASEB J. 35, e22039. 10.1096/fj.202100346R 34793600

[B143] Ryu H. Sun X. Chen Y. Li Y. Wang X. Dai R. S. (2021). The deubiquitinase USP36 promotes snoRNP group SUMOylation and is essential for ribosome biogenesis. EMBO Rep. 22, e50684. 10.15252/embr.202050684 33852194 PMC8183414

[B144] Saito K. Kigawa T. Koshiba S. Sato K. Matsuo Y. Sakamoto A. (2004). The CAP-Gly domain of CYLD associates with the proline-rich sequence in NEMO/IKKgamma. Structure 12, 1719–1728. 10.1016/j.str.2004.07.012 15341735

[B145] Sakamaki J. Mizushima N. (2023). Ubiquitination of non-protein substrates. Trends Cell Biol. 33, 991–1003. 10.1016/j.tcb.2023.03.014 37120410

[B146] Schechter I. Berger A. (1967). On the size of the active site in proteases. I. Papain. I. Papain. Biochem. Biophys. Res. Commun. 27, 157–162. 10.1016/S0006-291X(67)80055-X 6035483

[B147] Schulz S. Chachami G. Kozaczkiewicz L. Winter U. Stankovic-Valentin N. Haas P. (2012). Ubiquitin-specific protease-like 1 (USPL1) is a SUMO isopeptidase with essential, non-catalytic functions. EMBO Rep. 13, 930–938. 10.1038/embor.2012.125 22878415 PMC3463963

[B148] Shen L. N. Liu H. Dong C. Xirodimas D. Naismith J. H. Hay R. T. (2005). Structural basis of NEDD8 ubiquitin discrimination by the deNEDDylating enzyme NEDP1. EMBO J. 24, 1341–1351. 10.1038/sj.emboj.7600628 15775960 PMC1142549

[B149] Shi J. H. Sun S. C. (2018). Tumor necrosis factor receptor-associated factor regulation of nuclear factor κB and mitogen-activated protein kinase pathways. Front. Immunol. 9, 1849. 10.3389/fimmu.2018.01849 30140268 PMC6094638

[B150] Shin D. Mukherjee R. Grewe D. Bojkova D. Baek K. Bhattacharya A. (2020). Papain-like protease regulates SARS-CoV-2 viral spread and innate immunity. Nature 587, 657–662. 10.1038/s41586-020-2601-5 32726803 PMC7116779

[B151] Shin E. J. Shin H. M. Nam E. Kim W. S. Kim J. H. Oh B. H. (2012). DeSUMOylating isopeptidase: a second class of SUMO protease. EMBO Rep. 13, 339–346. 10.1038/embor.2012.3 22370726 PMC3321169

[B152] Shin J. Kim Y. H. Lee B. Chang J. H. Choi H. Y. Lee H. (2022). USP13 regulates HMGB1 stability and secretion through its deubiquitinase activity. Mol. Med. 28, 164. 10.1186/s10020-022-00596-0 36585612 PMC9801610

[B153] Shin Y. C. Tang S. J. Chen J. H. Liao P. H. Chang S. C. (2011). The molecular determinants of NEDD8 specific recognition by human SENP8. PLoS One 6, e27742. 10.1371/journal.pone.0027742 22110750 PMC3215745

[B154] Sorensen C. M. Rempel L. A. Nelson S. R. Francis B. R. Perry D. J. Lewis R. V. (2007). The hinge region between two ubiquitin-like domains destabilizes recombinant ISG15 in solution. Biochemistry 46, 772–780. 10.1021/bi061408x 17223698

[B155] Speer S. D. Li Z. Buta S. Payelle-Brogard B. Qian L. Vigant F. (2016). ISG15 deficiency and increased viral resistance in humans but not mice. Nat. Commun. 7, 11496. 10.1038/ncomms11496 27193971 PMC4873964

[B156] Sriramachandran A. M. Meyer-Teschendorf K. Pabst S. Ulrich H. D. Gehring N. H. Hofmann K. (2019). Arkadia/RNF111 is a SUMO-targeted ubiquitin ligase with preference for substrates marked with SUMO1-capped SUMO2/3 chain. Nat. Commun. 10, 3678. 10.1038/s41467-019-11549-3 31417085 PMC6695498

[B157] Stangl A. Elliott P. R. Pinto-Fernandez A. Bonham S. Harrison L. Schaub A. (2019). Regulation of the endosomal SNX27-retromer by OTULIN. Nat. Commun. 10, 4320. 10.1038/s41467-019-12309-z 31541095 PMC6754446

[B158] Suh H. Y. Kim J. H. Woo J. S. Ku B. Shin E. J. Yun Y. (2012). Crystal structure of DeSI-1, a novel deSUMOylase belonging to a putative isopeptidase superfamily. Proteins Struct. Funct. Bioinforma. 80, 2099–2104. 10.1002/prot.24093 22498933

[B159] Sun L. Gao J. Huo L. Sun X. Shi X. Liu M. (2010). Tumour suppressor CYLD is a negative regulator of the mitotic kinase Aurora-B. J. Pathology 221, 425–432. 10.1002/path.2723 20593489

[B160] Sun X. X. Challagundla K. B. Dai M. S. (2012). Positive regulation of p53 stability and activity by the deubiquitinating enzyme Otubain 1. EMBO J. 31, 576–592. 10.1038/emboj.2011.434 22124327 PMC3273389

[B161] Swaim C. D. Scott A. F. Canadeo L. A. Huibregtse J. M. (2017). Extracellular ISG15 signals cytokine secretion through the LFA-1 integrin receptor. Mol. Cell 68, 581–590. 10.1016/j.molcel.2017.10.003 29100055 PMC5690536

[B162] Swatek K. N. Komander D. (2016). Ubiquitin modifications. Cell Res. 26, 399–422. 10.1038/cr.2016.39 27012465 PMC4822133

[B163] Tatham M. H. Geoffroy M. C. Shen L. Plechanovova A. Hattersley N. Jaffray E. G. (2008). RNF4 is a poly-SUMO-specific E3 ubiquitin ligase required for arsenic-induced PML degradation. Nat. Cell Biol. 10, 538–546. 10.1038/ncb1716 18408734

[B164] Thibaudeau T. A. Anderson R. T. Smith D. M. (2018). A common mechanism of proteasome impairment by neurodegenerative disease-associated oligomers. Nat. Commun. 9, 1097. 10.1038/s41467-018-03509-0 29545515 PMC5854577

[B165] Tokunaga F. Nishimasu H. Ishitani R. Goto E. Noguchi T. Mio K. (2012). Specific recognition of linear polyubiquitin by A20 zinc finger 7 is involved in NF-κB regulation. EMBO J. 31, 3856–3870. 10.1038/emboj.2012.241 23032187 PMC3463848

[B166] Tracz M. Bialek W. (2021). Beyond K48 and K63: non-canonical protein ubiquitination. Cell Mol. Biol. Lett. 26, 1. 10.1186/s11658-020-00245-6 33402098 PMC7786512

[B167] Uchida N. Hoshino S. I. Katada T. (2004). Identification of a human cytoplasmic poly(A) nuclease complex stimulated by poly(A)-binding protein. J. Biol. Chem. 279, 1383–1391. 10.1074/jbc.M309125200 14583602

[B168] van den Heuvel J. Ashiono C. Gillet L. Dörner K. Wyler E. Zemp I. (2021). Processing of the ribosomal ubiquitin-like fusion protein FUBI-eS30/FAU is required for 40S maturation and depends on USP36. Elife 10, e70560. 10.7554/eLife.70560 34318747 PMC8354635

[B169] Van Der Veen A. G. Ploegh H. L. (2012). Ubiquitin-like proteins. Annu. Rev. Biochem. 81, 323–357. 10.1146/annurev-biochem-093010-153308 22404627

[B170] Van Leuken R. J. Luna-Vargas M. P. Sixma T. K. Wolthuis R. M. F. Medema R. H. (2008). Usp39 is essential for mitotic spindle checkpoint integrity and controls mRNA-levels of aurora B. Cell Cycle 7, 2710–2719. 10.4161/cc.7.17.6553 18728397

[B171] van Wijk S. J. Fulda S. Dikic I. Heilemann M. (2019). Visualizing ubiquitination in mammalian cells. EMBO Rep. 20, e46520. 10.15252/embr.201846520 30665942 PMC6362358

[B172] Verhelst K. Carpentier I. Kreike M. Meloni L. Verstrepen L. Kensche T. (2012). A20 inhibits LUBAC-mediated NF-κB activation by binding linear polyubiquitin chains via its zinc finger 7. EMBO J. 31, 3845–3855. 10.1038/emboj.2012.240 23032186 PMC3463847

[B173] Vijayasimha K. Dolan B. P. (2021). The many potential fates of non-canonical protein substrates subject to neddylation. Cells 10, 2660. 10.3390/cells10102660 34685640 PMC8534235

[B174] Vogl A. M. Phu L. Becerra R. Giusti S. A. Verschueren E. Hinkle T. B. (2020). Global site-specific neddylation profiling reveals that NEDDylated cofilin regulates actin dynamics. Nat. Struct. Mol. Biol. 27, 210–220. 10.1038/s41594-019-0370-3 32015554

[B175] Wagner K. Kunz K. Piller T. Tascher G. Hölper S. Stehmeier P. (2019). The SUMO isopeptidase SENP6 functions as a rheostat of chromatin residency in genome maintenance and chromosome dynamics. Cell Rep. 29, 480–494. 10.1016/j.celrep.2019.08.106 31597105

[B176] Walden M. Masandi S. K. Pawłowski K. Zeqiraj E. (2018). Pseudo-DUBs as allosteric activators and molecular scaffolds of protein complexes. Biochem. Soc. Trans. 46, 453–466. 10.1042/BST20160268 29472364

[B177] Walters B. J. Hallengren J. J. Theile C. S. Ploegh H. L. Wilson S. M. Dobrunz L. E. (2014). A catalytic independent function of the deubiquitinating enzyme USP14 regulates hippocampal synaptic short-term plasticity and vesicle number. J. Physiology 592, 571–586. 10.1113/jphysiol.2013.266015 PMC3934702 24218545

[B178] Wang T. Yin L. Cooper E. M. Lai M. Y. Dickey S. Pickart C. M. (2009). Evidence for bidentate substrate binding as the basis for the K48 linkage specificity of otubain 1. J. Mol. Biol. 386, 1011–1023. 10.1016/j.jmb.2008.12.085 19211026 PMC2682458

[B179] Wertz I. E. Newton K. Seshasayee D. Kusam S. Lam C. Zhang J. (2015). Phosphorylation and linear ubiquitin direct A20 inhibition of inflammation. Nature 528, 370–375. 10.1038/nature16165 26649818

[B180] Wickström S. A. Masoumi K. C. Khochbin S. Fässler R. Massoumi R. (2010). CYLD negatively regulates cell-cycle progression by inactivating HDAC6 and increasing the levels of acetylated tubulin. EMBO J. 29, 131–144. 10.1038/emboj.2009.317 19893491 PMC2775896

[B181] Wiener R. Dibello A. T. Lombardi P. M. Guzzo C. M. Zhang X. Matunis M. J. (2013). E2 ubiquitin-conjugating enzymes regulate the deubiquitinating activity of OTUB1. Nat. Struct. Mol. Biol. 20, 1033–1039. 10.1038/nsmb.2655 23955022 PMC3941643

[B182] Wiener R. Zhang X. Wang T. Wolberger C. (2012). The mechanism of OTUB1-mediated inhibition of ubiquitination. Nature 483, 618–622. 10.1038/nature10911 22367539 PMC3319311

[B183] Willingham S. Outeiro T. F. DeVit M. J. Lindquist S. L. Muchowski P. J. (2003). Yeast genes that enhance the toxicity of a mutant huntingtin fragment or alpha-synuclein. Science 302, 1769–1772. 10.1126/science.1090389 14657499

[B184] Wilson S. M. Bhattacharyya B. Rachel R. A. Coppola V. Tessarollo L. Householder D. B. (2002). Synaptic defects in ataxia mice result from a mutation in Usp14, encoding a ubiquitin-specific protease. Nat. Genet. 32, 420–425. 10.1038/ng1006 12368914

[B185] Wu J. Chen Y. Geng G. Li L. Yin P. Nowsheen S. (2019). USP39 regulates DNA damage response and chemo-radiation resistance by deubiquitinating and stabilizing CHK2. Cancer Lett. 449, 114–124. 10.1016/j.canlet.2019.02.015 30771428

[B186] Xie W. Jin S. Wu Y. Xian H. Tian S. Liu D. A. (2020). Auto-ubiquitination of NEDD4-1 recruits USP13 to facilitate autophagy through deubiquitinating VPS34. Cell Rep. 30, 2807–2819. 10.1016/j.celrep.2020.01.088 32101753

[B187] Yang S. Liu L. Cao C. Song N. Wang Y. Ma S. (2018). USP52 acts as a deubiquitinase and promotes histone chaperone ASF1A stabilization. Nat. Commun. 9, 1285. 10.1038/s41467-018-03588-z 29599486 PMC5876348

[B188] Yang W.-L. Wang J. Chan C.-H. Lee S.-W. Campos A. D. Lamothe B. (2009). The E3 ligase TRAF6 regulates akt ubiquitination and activation. Science 325, 1134–1138. 10.1126/science.1175065 19713527 PMC3008763

[B189] Yoo H. M. Kang S. H. Kim J. Y. Lee J. E. Seong M. W. Lee S. W. (2014). Modification of asc1 by ufm1 is crucial for erα transactivation and breast cancer development. Mol. Cell 56, 261–274. 10.1016/j.molcel.2014.08.007 25219498

[B190] Zhang S. Zou S. Yin D. Zhao L. Finley D. Wu Z. (2022). USP14-regulated allostery of the human proteasome by time-resolved cryo-EM. Nature 605, 567–574. 10.1038/s41586-022-04671-8 35477760 PMC9117149

[B191] Zhang T. Ma C. Zhang Z. Zhang H. Hu H. (2021). NF-κB signaling in inflammation and cancer. MedComm (Beijing) 2, 618–653. 10.1002/mco2.104 PMC8706767 34977871

[B192] Zhang X. Choi P. S. Francis J. M. Imielinski M. Watanabe H. Cherniack A. D. (2016). Identification of focally amplified lineage-specific super-enhancers in human epithelial cancers. Nat. Genet. 48, 176–182. 10.1038/ng.3470 26656844 PMC4857881

[B193] Zhao Q. Pavanello L. Bartlam M. Winkler G. S. (2023a). Structure and function of molecular machines involved in deadenylation-dependent 5′-3′ mRNA degradation. Front. Genet. 14, 1233842. 10.3389/fgene.2023.1233842 37876592 PMC10590902

[B194] Zhao Y. Ma C. A. Wu L. Iwai K. Ashwell J. D. Oltz E. M. (2015a). CYLD and the NEMO zinc finger regulate tumor necrosis factor signaling and early embryogenesis. J. Biol. Chem. 290, 22076–22084. 10.1074/jbc.M115.658096 26224629 PMC4571959

[B195] Zhao Y. Majid M. C. Soll J. M. Brickner J. R. Dango S. Mosammaparast N. (2015b). Noncanonical regulation of alkylation damage resistance by the OTUD 4 deubiquitinase. EMBO J. 34, 1687–1703. 10.15252/embj.201490497 25944111 PMC4475402

[B196] Zhao Z. O’Dea R. Wendrich K. Kazi N. Gersch M. (2023b). Native semisynthesis of isopeptide-linked substrates for specificity analysis of deubiquitinases and ubl proteases. J. Am. Chem. Soc. 145, 20801–20812. 10.1021/jacs.3c04062 37712884 PMC10540217

[B197] Zhou Y. Zhu Y. (2015). Diversity of bacterial manipulation of the host ubiquitin pathways. Cell Microbiol. 17, 26–34. 10.1111/cmi.12384 25339545

[B198] Zhu Q. Fu Y. Cui C. P. Ding Y. Deng Z. Ning C. (2023). OTUB1 promotes osteoblastic bone formation through stabilizing FGFR2. Signal Transduct. Target Ther. 8, 142. 10.1038/s41392-023-01354-2 37024477 PMC10079838

